# Transposable element expression in tumors is associated with immune infiltration and increased antigenicity

**DOI:** 10.1038/s41467-019-13035-2

**Published:** 2019-11-19

**Authors:** Yu Kong, Christopher M. Rose, Ashley A. Cass, Alexander G. Williams, Martine Darwish, Steve Lianoglou, Peter M. Haverty, Ann-Jay Tong, Craig Blanchette, Matthew L. Albert, Ira Mellman, Richard Bourgon, John Greally, Suchit Jhunjhunwala, Haiyin Chen-Harris

**Affiliations:** 1 Department of Genetics and Center for Epigenomics, Albert Einstein College of Medicine, New York, 10461 USA; 2 Genentech, Inc., 1 DNA Way, South San Francisco, CA 94080 USA; 3 Department of Bioinformatics Interdepartmental Program, University of California at Los Angeles, Los Angeles, CA USA; 4 Argonaut Genomics, Inc., 1025 Alameda De Las Pulgas #806, Belmont, CA 94002 USA

**Keywords:** Cancer genomics, Immunotherapy

## Abstract

Profound global loss of DNA methylation is a hallmark of many cancers. One potential consequence of this is the reactivation of transposable elements (TEs) which could stimulate the immune system via cell-intrinsic antiviral responses. Here, we develop *REdiscoverTE*, a computational method for quantifying genome-wide TE expression in RNA sequencing data. Using The Cancer Genome Atlas database, we observe increased expression of over 400 TE subfamilies, of which 262 appear to result from a proximal loss of DNA methylation. The most recurrent TEs are among the evolutionarily youngest in the genome, predominantly expressed from intergenic loci, and associated with antiviral or DNA damage responses. Treatment of glioblastoma cells with a demethylation agent results in both increased TE expression and de novo presentation of TE-derived peptides on MHC class I molecules. Therapeutic reactivation of tumor-specific TEs may synergize with immunotherapy by inducing inflammation and the display of potentially immunogenic neoantigens.

## Abstract (web summary)

Treatment with demethylation agents can reactivate transposable elements. Here in glioblastoma, the authors also show that this is accompanied by de novo presentation of TE-derived peptides on MHC class I molecules.

## Introduction

Over the past few years, we have come to understand that tumor-associated neoantigens provide important targets for anticancer T-cell responses. This conceptual breakthrough has led to efforts to therapeutically target patient-specific mutations using personalized vaccines^1^. Neoantigens stemming from point mutations in the coding exons alone, however, likely underestimate the true mutational burden in the tumor^2,3^; other cancer-specific antigens might also exist and contribute to immune response against the tumor. Indeed, there is growing evidence from in vitro and preclinical studies that DNA demethylation inhibitors can trigger innate antiviral response to human endogenous retroviral (HERVs) expression in the tumor^4–7^. Such retroviral transcripts also have the potential to produce antigens that activate adaptive immunity^8^. HERVs, together with other classes of transposable elements (TEs) comprise about 45% of the human genome, a sequence space that vastly eclipses that of the coding genome^9^. Although in normal tissue, much of TE activity is under tight epigenetic control, in cancer, we hypothesized that wide-spread TE expression occurs, particularly in those with extensive epigenetic dysregulation^10^. Investigation of the extent of expression by these evolutionarily sequestered sequences in cancer, and their antigenic potential, has thus far been very limited.

A major impediment to understanding TE expression and its potential relevance to tumor immunity is the analytic challenge of accurate quantification of short-read sequences from repetitive regions in the transcriptome. Standard pipelines typically discard repetitive reads^11^. To shed light on these hidden parts of the transcriptome, we have developed and benchmarked a new method, *REdiscoverTE*, to comprehensively quantify expression by all repetitive elements including TEs in RNA-seq data, then applied it to 7750 cancer and normal tissue samples from The Cancer Genome Atlas (TCGA) and a replication study^12^. Here, we provide the first landscape analysis of TE expression in cancer, reveal that the rate of tumor DNA demethylation *proximal* to TEs is far greater than global demethylation, and identify multiple ways in which TE expression may impact cellular and immune responses to the tumor. We show that tumor cells present potentially immunogenic peptides derived not only from HERVs, but also other classes of TE: long interspersed nuclear elements (LINE), short interspersed nuclear elements (SINE), and SINE-VNTR-Alu (SVA). TE-derived tumor-specific antigens that are conserved and not patient-specific may be evaluated as “off-the-shelf” vaccine targets both for therapeutic intervention and, even more provocatively, for cancer prophylaxis.

## Results

### Genome-wide TE expression quantification approach

**Fig. 1:**
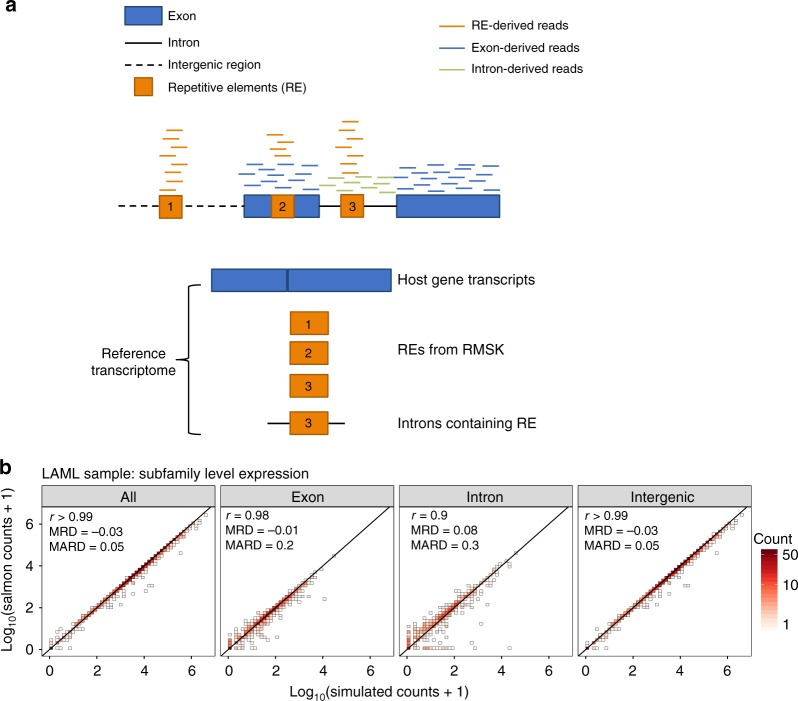
*REdiscoverTE* reference transcriptome and performance benchmarking. **a** REdiscoverTE whole-transcriptome reference for RE quantification. Schematic depicts short reads mapping to host gene features (exons, introns) and REs either embedded within host genes or intergenic regions. RE genomic locations are derived from RepeatMasker (RMSK). Reads stemming from repetitive elements (REs), exons, and introns are illustrated in orange, blue and green, respectively. **b** Benchmarking the accuracy of RE quantification by REdiscoverTE with simulation. Two-dimensional histogram comparing REdiscoverTE quantification to simulated RE expression generated based on a TCGA LAML sample. Expression is aggregated to the subfamily level. Left to right: all RE expression regardless of genomic context, exonic RE expression, intronic RE expression, intergenic RE expression. Performance accuracy is measured in terms of Spearman correlation coefficient (r), mean relative difference (MRD), mean absolute relative difference (MARD)

*REdiscoverTE* was devised to simultaneously quantify expression by all annotated genes defined in Gencode^13^ and all RepeatMasker sequences (over five million) in the human genome^14^ (Fig. 1a, Supplementary Table 1, detailed in the Methods). To mitigate the uncertainty associated with mapping reads to repetitive features, we leveraged a recently developed light-weight mapping approach for isoform quantification, *Salmon*, which uses an expectation-maximization (EM) algorithm to assign multi-mapping reads probabilistically to transcripts, based on evidence from uniquely mapped reads^15^. Post-quantification, we restricted our downstream analysis to only TEs sequences (over four million), which are classified into 1052 distinct TE subfamilies in five classes: LINE, SINE, long terminal repeats (LTR), SVA, and DNA transposons (Supplementary Fig. 1a). To measure the total transcriptional output from a group of related TEs, we aggregated expression for individual elements to the TE subfamily level. To further distinguish autonomous TE expression from co-expression with host genes or intron retention, we next divided the aggregated expression for each TE subfamily into exonic, intronic, and intergenic expression by stratifying all elements under a given TE subfamily by their genomic locations with respect to host genes. For example, of the 1610 annotated instances of *L1HS*, 951, 654, and 5 are located in the intergenic regions, gene intronic regions and gene exons, respectively. Here, *L1HS* intergenic expression was defined as the aggregate expression from the 951 elements within the intergenic regions. Other subfamilies were treated similarly.

We benchmarked the performance of *REdiscoverTE* with extensive simulations using *RSEM*^16^ (Fig. 1b, Supplementary Fig. 1). *REdiscoverTE’s* approach of expression aggregation to the subfamily level demonstrated high accuracy of TE quantification (Spearman correlation *r* ≥ 0.99, mean absolute relative difference, mean absolute relative difference (MARD) ≤ 0.05), likely by reducing mapping noise observed at the individual element level (Supplementary Fig. 1e–f). *REdiscoverTE* performed best on intergenic TEs, followed by exonic, then intronic TEs (Fig. 1b, Supplementary Fig. 1f). Exonic TE expression, which comprised a minority of the total TE read fraction, was excluded from further analysis to rule out the potential confounding expression from overlapping host genes and ease downstream interpretation.

We compared *REdiscoverTE* to three previously published TE expression quantification methods: the method used in^6^, *RepEnrich*^17^, and *SalmonTE*^18^ (Supplementary Fig. 1g–k, Supplementary Note 1) and have found *REdiscoverTE* to be significantly more comprehensive and accurate as a robust, whole-transcriptome quantification method.

### TE expression is dysregulated in cancer

To characterize the landscape of TE expression in cancer, we applied *REdiscoverTE* to 7345 TCGA RNA-seq samples (containing 1232 tumor and matched normal samples, the rest are tumor samples without normals) across 25 cancer types. For validation of our findings in select cancer types, we also analyzed an additional 405 tumor and matched normal RNA-seq samples across five cancer types (Supplementary Table 2) from the Cancer Genome Project (CGP)^12^. Both data sets were generated from poly-dT RNA-seq library preparations, which can capture poly-adenylated TEs transcripts. On average, 1% of RNA-seq output mapped to TEs (Supplementary Fig. 2a). Notably, TE expression was observed from all TE classes (*N* = 5) and most families (*N* = 43), including both retrotransposons and DNA transposons (Supplementary Fig. 2b). For each TE class, the bulk of expression stems from intergenic regions (Supplementary Fig. 2c), suggesting autonomous TE expression from intergenic loci compared with read-through transcription in host, protein-coding genes. Human DNA transposons had been thought to be completely inactive, based on the lack of evidence for transposition in the human genome^19^. Although these data cannot address transposition, our results suggest active gene expression by DNA transposons in many cancers.

**Fig. 2:**
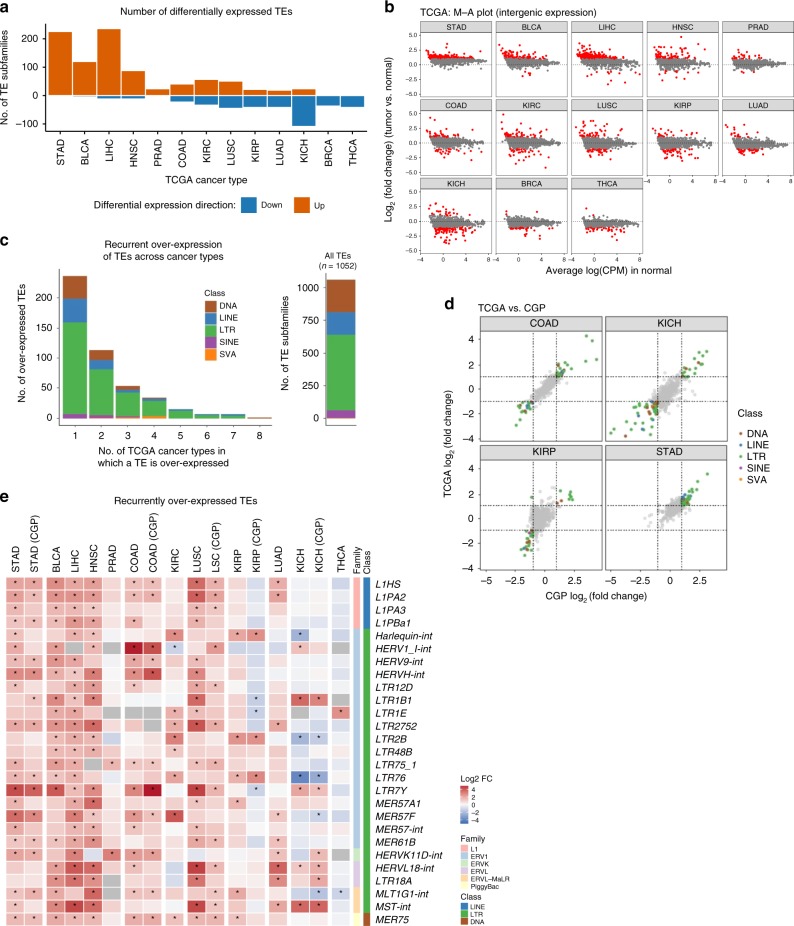
TE expression is dysregulated in cancer. **a** Number of differentially expressed TE subfamilies in 13 TCGA cancer types. Red bars: number of significantly overexpressed TE subfamilies. Blue bars: number of significantly underexpressed TE subfamilies. Cancer types are ordered from left to right by the ratio between number of overexpressed and underexpressed TE subfamilies. Differential expression analyses are based on intergenic TE expression and performed on matched tumor-normal sample pairs. Significance is defined at log2 fold change (FC) > 1 and FDR < 0.05. **b** M-A plot showing TE expression FC of tumor over normal as a function of mean normal tissue TE expression (log2 counts per million; CPM) for 13 TCGA cancer types. Each point is one TE subfamily. Red: significantly differentially expressed TE subfamilies. Cancer types are ordered as in Fig. 2a. **c** Left: histogram of TE subfamilies by number of TCGA cancer types in which they are overexpressed to show recurrence of overexpression. In total, 27 TE subfamilies were overexpressed in at least five cancer types. Right: number of TE subfamilies in each of five TE classes as defined by Repeatmasker GRCh38. **d** Comparison of TE differential expression profile (tumor vs. matched normal) between TCGA and CGP RNA-seq data on matching cancer types. **e** FC of expression for the 27 TE subfamilies are selected based on Fig. 2c. Heatmap colors indicate log2 FC (tumor vs. matched normal) values; columns are ordered as in Fig. 2a. CGP data are grouped with corresponding TCGA cancer types. *significant differential expression defined in **a**

In all cancer types, TE expression was detected in both tumor and matched normal tissues, suggesting basal levels of TE transcriptional activities in normal tissues. Across the two data sets, 10 cancer types showed a significantly higher proportion of reads mapping to TEs in tumor compared with matched normal tissues, suggesting particularly active TE expression in these cancers; the reverse was observed in four cancer types (Supplementary Fig. 2a, d, e). Differential expression analysis of tumor samples with respect to matched normals^20–22^ confirmed that stomach, bladder (Supplementary Fig. 2f), liver, and head and neck tumors show predominantly overexpression of TEs, whereas thyroid, breast, kidney chromophobe, and lung adenocarcinoma tumors show predominantly reduced TE expression compared with normal (Fig. 2a). Across all tumor types, many TEs showed differential expression: out of 1052 TE subfamilies, 587 were differentially expressed in at least one TCGA cancer type studied, of which, 463 are overexpressed in at least one cancer type (Fig. 2a–c, Supplementary Data 1, Supplementary Data 2). The TE class LTR showed the highest number of overexpressed subfamilies followed by DNA and LINE. The pattern of tumor-over-normal fold change of expression was highly consistent across the two data sets (TCGA and CGP), suggesting that the TE expression profile may be characteristic of tumor/tissue type (Fig. 2d).

A minority of 61 TE subfamilies were significantly and recurrently overexpressed in at least four cancer types (Fig. 2c). Among these, *MER75*, a member of the piggyBac DNA transposon^9^ and *L1HS*, the human specific subfamily of the LINE1 family, were overexpressed in at least eight cancer types (TCGA and CGP combined, Fig. 2e). Many subfamilies belonging to the ERV1, ERVK, ERVL, and ERVL-MaLR family were also recurrently overexpressed in cancer. Full-length HERV sequences consist of two LTRs flanking a proviral genome^19,23^. *HERVH-int*, the proviral portion of *HERVH* and *LTR7Y*, the youngest variant of the LTRs associated with *HERVH*^24^, were simultaneously overexpressed within patients in six cancer types (Fig. 2e, Supplementary Fig. 2g, h, Supplementary Data 2), suggesting either strong co-regulation or possible expression of full-length HERVH sequence. A similar pattern of co-expression was also found in *HERVL18-int* and its associated LTR element *LTR18A*, and to some extent *L1HS* and *SVA_F* (Supplementary Fig. 2h).

Overall, the most recurrently overexpressed TEs were among the evolutionarily youngest in the human genome: piggyBac, *L1HS, HERVK,* and *HERVH*. This trend is also observed within the well-defined *L1PA* lineage (Supplementary Data 2), where *L1HS* (a.k.a. *L1PA1*) and *L1PA2*—the two youngest L1 subfamilies—were overexpressed in the most number of TCGA cancer types (*n* = 7), followed by fewer recurrences by the increasingly older subfamilies of *L1PA3* (*n* = 5 cancer types), *L1PA5* (*n* = 4), and *L1PA8* (*n* = 3). This trend is not strictly replicated in other well-defined TE families. For example, the six SVA subfamilies are named A–F in the order from the oldest to the youngest. In TCGA data, we observed that *SVA_A*, *SVA_B*, and *SVA_F* were recurrently overexpressed in four cancer types, whereas *SVA_D*, *SVA_E*, and *SVA_C* were overexpressed in 2–3 cancer types (Supplementary Data 2). Nevertheless, younger TEs may have better transcriptional potential in tumor genomes owing either to more intact sequences and thus preserved promotors or to fewer overlapping mechanisms of silencing.

### TE expression is associated with proximal DNA demethylation

**Fig. 3:**
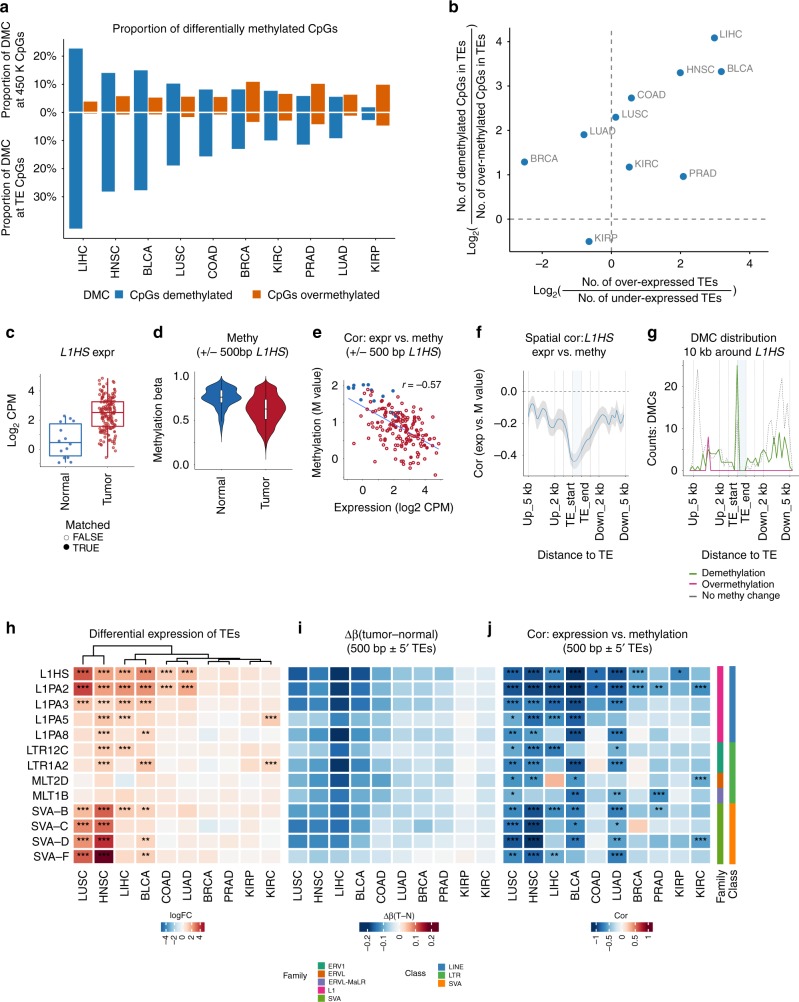
TE expression in cancer is associated with epigenetic dysregulation. **a** Global differential methylation states across TCGA cancer types. Criteria for significant DMCs: absolute (Δbeta) ≥ 10%, FDR < 0.05. Top: proportion of DMCs among all Illumina 450 K CpG sites. Bottom: proportion of DMCs at CpGs within TEs. Blue: proportion of demethylated DMCs among all CpG sites. Orange: proportion of over-methylated DMCs. **b** TE mRNA overexpression correlation with the extent of CpG demethylation within TEs. Each point represents one cancer type. Horizontal axis: log2 ratio between the number of overexpressed TE subfamilies and the number of underexpressed TE subfamilies. Vertical axis: log2 ratio between the number of demethylated DMCs in TEs and the number of over-methylated DMCs in TEs. **c**–**g** Association between *L1HS* intergenic expression and its DNA methylation state in BLCA (all samples). **c** *L1HS* intergenic expression in normal and tumor samples. Blue: normal sample. Red: tumor samples. Filled circle: tumor samples with matched normal. Open circle: tumor samples without matched normal). **d** *L1HS* proximal CpG *M* value in normal and tumor samples. Blue: normal samples. Red: tumor samples. CpG sites are from 500 bp ± regions around intergenic *L1HS* 5′ bp. **e** Pearson correlation between intergenic *L1HS* expression and methylation *M* value. **f** Spatial correlation between *L1HS* expression and CpG methylation *M* value 5 kb ± *L1HS*. Correlation was calculated for all samples at each CpG site, then smoothed with binsize = 500 bp. Shading indicates 95% confidence interval. **g** Spatial distribution of demethylated CpG (green), over-methylated CpGs (red) and CpGs with no methylation change (gray, dashed) 5 kb ± around *L1HS*. Binsize = 500 bp. **h**–**j** Examples of selected TE subfamilies with significant negative correlation (Spearman cor ≤ −0.4 & FDR < 0.05) between intergenic expression and methylation in more than four types of tumors (based on matched samples only). **h** Tumor vs. Normal differential expression. Heatmap colors: log2 FC. Significance level *: logFC > 1 & FDR < 0.05; **: logFC > 1 & FDR < 0.01; ***: logFC > 1 & FDR < 0.001. **i** Tumor-normal average Δbeta in 500 bp ± regions around 5′ bp of all intergenic loci of given TE subfamily. **j** Correlation between intergenic TE expression and *M* values ~ 500 bp ± 5′ bp of intergenic TE. Heatmap colors: correlation (cor) coefficient. Significance level *: abs(cor) ≥ 0.4&FDR < 0.05, **: abs(cor) ≥ 0.4&FDR < 0.01; ***: abs(cor) ≥ 0.4 & FDR < 0.001

The human genome encodes multiple defense strategies to silence the expression and mobility of TEs in germ cells and normal tissues, including epigenetic repression by DNA methylation^25–27^. In cancer, however, cellular transformation to a malignant state is frequently accompanied by a global loss of DNA methylation^10,28^. To elucidate the role of DNA methylation alterations in TE expression, we examined in 10 TCGA cancer types DNA methylation changes from normal to tumor at both the genome-wide (global) and TE-proximal level using TCGA Illumina 450 K array data, which captured 70 K CpG sites overlapping with 1007 TE subfamilies (Supplementary Fig. 3a). At the global level, we observed strong CpG demethylation of tumor tissue compared with normal in liver, head and neck, bladder and lung squamous and colon cancers (Supplementary Fig. 3b), where the majority of differentially methylated CpGs (DMCs) in these cancer types were demethylated (Δbeta < −0.1, FDR < 0.05, Fig. 3a top, Supplementary Fig. 3c, Supplementary Table 3). However, in all 10 cancer types considered, we discovered a striking enrichment of demethylated DMCs located within TEs as compared with background level of DNA demethylation on the 450 K array (Fig. 3a bottom vs. top), suggesting that a greater rate of loss of DNA methylation at TE regions may be a common tumor pathology. Consistent with the known role of DNA methylation for TE silencing, the extent of demethylation, in terms of log-ratio of demethylated vs. over-methylated TE CpGs, was strongly associated with the extent of TE overexpression across cancer types (Fig. 3b).

To gain better resolution on methylation patterns around TEs, we performed sample-level correlation and spatial analysis of DMCs. We illustrate the approach with intergenic *L1HS* expression and methylation analysis in BLCA. Relative to normal bladder tissue, *L1HS* is significantly overexpressed in bladder tumor (log2 FC = 2.3, *p* = 4 × 10^−7^, *t* test, Fig. 3c); and an inverse relationship is seen for methylation marks, with *L1HS* proximal (± 500 bp) CpG sites being significantly demethylated in tumor compared with normal tissue (*p* = 2 × 10^−16^, two sided *t* test, Fig. 3d). Across samples, the average *L1HS* methylation level was significantly inversely correlated with aggregate *L1HS* expression level (Pearson cor = −0.57, *p* = 2 × 10^−15^, Fig. 3e). Next we created a spatial correlation profile between methylation level for a 10 kb region around *L1HS* and aggregated expression level and observed a deepening inverse correlation at the 5′ end of *L1HS* in BLCA (Fig. 3f). Finally, a DMC spatial enrichment profile for the same 10 kb region further confirmed a strong enrichment of demethylated DMCs at the 5′ end of *L1HS* in BLCA (Fig. 3g). These results together establish that intergenic *L1HS* activity in tissue is influenced by the DNA methylation state at its 5′ end.

We extended this correlation analysis to 1007 TE subfamilies in 10 cancer types and discovered a strong inverse correlation for 431 TE subfamilies (Supplementary Fig. 3d, Supplementary Data 3), 262 of which showed significant overexpression in at least one cancer type. We highlight 13 TEs subfamilies from the LINE, LTR, and SVA class that showed recurrent significant inverse correlation between expression and proximal DNA methylation across cancer types (Fig. 3h–j). Of note, we found that the overexpression of SVA, the youngest active group of retroelements in hominids^29^, is strongly associated with proximal DNA demethylation, particularly in head and neck squamous cell and lung squamous cell carcinoma (Fig. 3j, Supplementary Fig. 3f).

As noted above, we observed predominantly reduced levels of TE expression in tumor compared with normal tissue in a subset of cancer types (e.g., breast and lung adenocarcinoma). We examined DNA methylation status at six recurrently downregulated TEs but found no clear association between methylation and TE expression (Supplementary Fig. 3e).

Together, these data demonstrate that the overexpression of many TEs in tumor is associated with loss of DNA methylation, particularly at TE-proximal CpG sites, suggesting that a major mechanism of TE expression may be targeted loss of DNA methylation near TEs.

### TE expression correlates with DNA damage and immune response

We next tested the hypothesis that tumor TE expression can impact cellular and immune response within the tumor by examining its relationship to transcriptional activities of major cellular pathways. Twenty-four pathways of biology were considered, including eight related to cancer (e.g., *P53* signaling), six related to DNA damage response (DDR) (e.g., homologous recombination) and eight related to immune response (e.g., type I IFN response)^30^ (Supplementary Data 4). For each pathway of interest, we first scored its overall activity in the tumor samples using singular value weighted gene expression of the associated geneset. To isolate the contribution of TE to variable expression of these pathways (relative to other factors such as immune infiltrate), we compared three lasso regularized regression models^31^: (1) a cellularity-only model that relates pathway scores to estimates of sample tumor purity plus lymphoid and myeloid cell content^32^ (Supplementary Fig. 4a, b); (2) a cellularity + true TE data model that relates pathway scores to the expression of 1052 TE subfamilies in addition to the three tumor cellular components (tumor, lymphoid, myeloid), and (3) a cellularity + permuted TE data model, where TE data have been scrambled sample-wise, whereas the correlation structures and the total number of predictors are preserved compared with model 2. The difference between the goodness of fit (the *r*^2^ values) of model 2 and model 1 was interpreted as the total contribution from all TE expression to each pathway (variance explained). As Lasso is a statistical technique that selects representatives of correlated variables, the models also helped to identify representative top TE contributors to the variable expression of each pathway in question (Supplementary Data 5). Model 3 was used to evaluate whether adding 1052 predictors that are random noise improved model performance. As expected, the performance of the permuted model did not improve with respect to the cellularity-only model, thus providing statistical confidence that any performance improvement observed in true TE model is not simply an artifact of additional predictors.

**Fig. 4:**
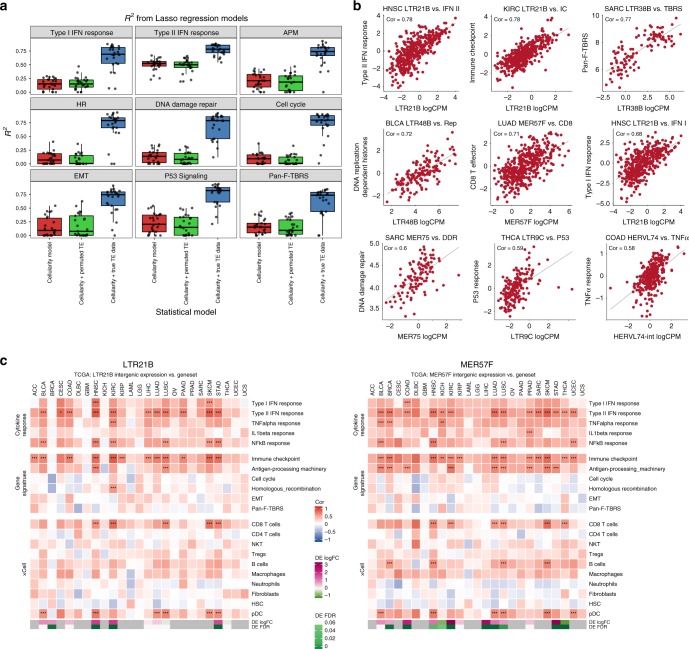
TE activity is associated with DNA damage and immune response in the tumor. **a** Comparison of *R*^2^ results of the three lasso models for nine gene signature scores. Each panel is one gene signature, each point is one of 25 cancer type. HR: homologous recombination. APM: antigen processing machinery. EMT: epithelia-mesenchymal-transition. Pan-F-TBRS: pan-fibroblast *TGFbeta* response signature. Red: *R*^2^ from the cellularity-only lasso models. Green: *R*^2^ from the cellularity + permuted TE models. Blue: R2 from the cellularity + true TE data models. **b** Examples of positive correlations between gene signature scores and TE expression levels in different TCGA cancer types. Each point is one tumor sample, gray line is the best fit from linear model. Cor: Spearman correlation coefficient. Gene signature scores were adjusted by tumor content using linear regression. **c** Association heatmap between one TE subfamily and multiple gene signatures and estimated immune infiltrates across 25 TCGA cancer types. Left: LTR21B. Right: MER57F. Color: Spearman correlation coefficient (cor) from partial correlation adjusting for tumor purity. Significance of correlation: * abs(cor) > 0.5 & FDR < 0.05, ** abs(cor) > 0.5 & FDR < 0.01; *** abs(cor) > 0.5 & FDR < 0.001. Bottom bars show the differential expression log2 fold change and FDR values of TE in each cancer type. Magenta: upregulated. Green: downregulated. Gray: either no normal samples available or the TE expression level was too low for a given cancer type

Comparing the cellularity + true TE model with the cellularity-only model revealed that total TE expression explained substantial fractions of variance in many gene expression signatures, including DDR, type I IFN response, antigen processing pathways, cell cycle, *P53* signaling, epithelial–mesenchymal transition, *WNT* target, and pan-fibroblast *TGFb* response, with mean changes in *R*^2^ values ranging 0.4–0.6 (Fig. 4a, Supplementary Fig. 4c, Supplementary Data 5). Specifically for pathways relevant to DDR (averaging over six signatures: homologous recombination, mismatch repair, nuclear excision repair, nonhomologous end joining, Fanconi anemia and DDR), the mean *R*^2^ values across cancer types was substantially higher in the cellularity + true TE data model (0.67 ± 0.24) than the cellularity-only model (0.09 ± 0.10). *MER75* (piggyBac DNA transposon), *MER4A* (ERV1 family), *MER54A* (ERV3), and *MER67A* (ERV1) were identified by the true TE model as top predictors for DDR activities (Fig. 4b, Supplementary Fig. 4d, e).

For type I IFN response expression, the mean *R*^2^ values across cancer types was 0.64 ± 0.23 in the cellularity + true TE model compared with 0.15 ± 0.10 in the cellularity-only model. *MamGypLTR2b* (Gypsy), *THE1C-int* (ERVL), *LTR21B* (ERV1), and *MER57F* (ERV1) were identified as TEs with the strongest positive association to type I IFN response (Fig. 4b, c, Supplementary Fig. 4d). Type I IFN response is indicative of activation of cell-intrinsic antiviral pathways and has been suggested to be induced by intracellular sensing of dsRNA formed from TE transcripts^4,5^. Our models quantified the extent to which total TE expression contributes to type I IFN signals in different types of cancer. Direct correlation analysis with estimated tumor immune infiltrates also revealed for several cancer types positive association of *LTR21B* and *MER57F* to tumor plasma dendric cell (pDC) expression (Fig. 4c), which is consistent with known biological function of pDC as a potent producer of type I IFN.

Several HERV subfamilies, *LTR21B*, *MER57F,* and *HERVL74-int* (ERVL), were also identified as the top TE correlates to gene expression levels of type II IFN response, CD8 T effector and immune checkpoint activity inferred from tumor bulk tissue. Direct correlation analysis with estimated immune infiltrates confirmed positive association of *LTR21B* and *MER57F* to CD8 + T cells expression (Fig. 4c).

Given that total TE expression accounts for significant variance in DDR and immune response pathways, we next explored the directionality and strength of association from all individual TE subfamilies to these two biological systems using standard correlation analysis (Supplementary Fig. 4f). We identified striking positive correlations with DDR from a large number of TE subfamilies in renal clear cell carcinoma (KIRC, 327 subfamilies), pancreatic adenocarcinoma (PAAD, 111 subfamilies) and sarcoma (SARC, 51 subfamilies). KIRC, a highly immunogenic type of tumor, was recently found to harbor the highest proportion of insertion-and-deletion tumor mutations compared with other TCGA cancer types^2^. As the overall level of DDR-related expression in KIRC is comparable to many cancer types (Supplementary Fig. 4g), one possible explanation for the extensive positive correlations in KIRC is that TEs may be a significant contributor to DDR. Somatic TE transposition events have been previously described in TCGA samples to lead to insertional mutations private to tumors^33^ and retrotransposition is known to create DNA double-strand breaks^34^. Although DDR is known to activate immune signaling and inflammation^35^, more research is needed to elucidate the connection between TE expression, DDR and immunogenicity in cancer.

### Decitabine increases TE peptide expression in GBM cell lines

**Fig. 5:**
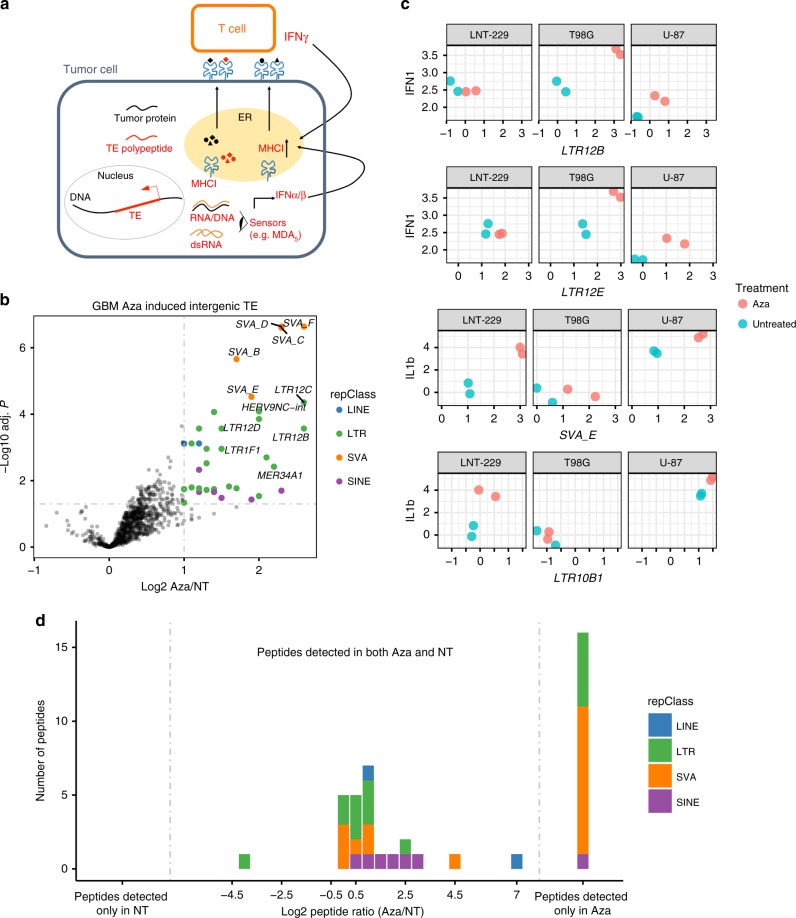
Decitabine increases TE expression and peptide presentation in GBM cell lines. **a** Working model of the impact of TE expression in the tumor. TE expression in the cytoplasm may trigger intracellular sensing of TE mRNA and result in type I IFN response. TE may be a source of tumor-associated antigens that can be presented at the tumor cell surface and recognized by TE-antigen specific T cells. **b** Volcano plot showing differential intergenic expression of TE subfamilies, Aza (decitabine) vs. NT (non-treated). TE subfamilies are colored by class at the significance threshold of log2 FC > 1 and BH-adjusted *p* value < 0.05 and labeled if log2 FC > 1.5 and adjust *p* value < 0.01. **c** Association between select overexpressed TE subfamilies and cytokine gene signatures. **d** Effect of decitabine treatment on TE peptide presentation. Middle panel: histogram on log2 FC for TE peptides abundance TE subfamilies with overexpression of mRNA. The log2 FC of peptide presentation was calculated by comparing spectral areas for each peptide in both Aza vs. NT conditions. Peptides detected only in Aza: peptides uniquely detected in the treated condition. No TE peptides were detected only in the NT condition

In addition to strong association to innate immune activation in the tumor, TE expression may also contribute to the adaptive immune infiltration by providing tumor cell surface antigens (Fig. 5a). Certain HERV transcripts have been shown to result in MHC class I-bound peptides at tumor cell surface and serve as triggers for cytolytic T-cell response^36,37^. We postulated that a variety of TE peptides may be presented by tumor and subject to surveillance by the adaptive immune system.

To pursue this question, we examined a data set consisting of matched transcriptome, proteome, and MHC class I peptidome data^38^ in which the authors originally examined the treatment effect of 5-aza-2’-deoxycytidine (decitabine, an inhibitor of DNA methyltransferase 1) on GBM cell lines. Applying *REdiscoverTE* to the GBM transcriptome generated with rRNA depletion library prep, we discovered a much higher proportion of TE transcripts (~2.5%) and an enrichment of intronic TE expression in comparison with the data from poly-dT library preps, independent of treatment group (Supplementary Fig. 5a, b). Epigenetic de-repression by decitabine resulted in strong overexpression of TEs originating from intergenic and intronic regions in these GBM cell lines, particularly TEs from SVA (SVA_B, C, D, E, F), ERV1 (*n* = 27), L1 (*n* = 2), and Alu (*n* = 9) families (FDR < 0.05 and FC > 2, Fig. 5b, Supplementary Fig. 5c, Supplementary Data 6).

We searched the matching MHC class I peptidome and whole proteome data for translational products of TE by performing peptide identification and label-free quantification based on an augmented human proteome that included TE sequences from 51 overexpressed TE subfamilies. Using this approach, we identified 83 unique MHC-presented peptides derived from TEs and chose a subset of 39 peptides that were detected at least three times across all samples for further analysis. The majority of peptides mapped to TE elements resided in the intergenic regions of the reference genome, and some mapped to intronic regions (Supplementary Data 7). Besides peptides derived from LTRs (*n* = 13), we also detected peptides derived from other TE classes: SVA (*n* = 17), SINE (*n* = 7), and LINE (*n* = 2), which have not been reported before. Two subfamilies (SVA_D and LTR12C) representing half of the 39 peptides. In addition, we identified 19 peptides derived from five of these subfamilies within the whole proteome data, adding further confidence in the translation potential of these TEs (Supplementary Data 8). Among the 39 MHC-I TE peptides, 16 (41%) were detected only in the decitabine-treated condition, suggesting increased presentation of possibly novel peptides induced by a 5-aza-2’. Seven of the remaining 23 peptides showed a twofold increase in abundance under decitabine treatment compared with untreated condition (Fig. 5c). A subset of the peptides detected under both treated and untreated conditions were synthesized; we were able to verify their identity by mass spectrometry (MS) and confirm binding of 26 peptides to HLA*03:01 by liquid chromatography MS peptide exchange assay (Supplementary Data 7).

Simultaneous to TE expression increase, decitabine treatment also resulted in a striking induction of host gene expression, including many cancer testis antigen (CTA) genes such as the *MAGE* family genes, *CYP1B1* and *MELTF* (Supplementary Fig. 5d), as also reported in Shraibman et al.^38^. Geneset enrichment analysis confirmed that decitabine treatment is associated with the overexpression of not only CTA related pathways–spermatogenesis (FDR = 5.1 × 10^−3^), allograft rejection (FDR = 1.3 × 10^−2^), but also a number of immune and cellular pathways that is consistent with above TCGA finding: inflammatory response (FDR = 4.0 × 10^−7^), *TNFα* signaling (FDR = 1.5 × 10^−3^), EMT (FDR = 6.4 × 10^−2^), and *P53* response (FDR = 0.03, Supplementary Data 9). Also consistent with above TCGA results are the observations that the expression of several TE subfamilies correlated with either IL1beta response or type I interferon response (Fig. 5b). Interestingly, expression of two subfamilies with the most number of peptides detected: *SVA_D* and *LTR12C*, were both strongly associated with DNA damage repair and homologous recombination in the TCGA KIRC cohort (*SVA_D* Spearman cor > 0.58, FDR < 4e-36, *LTR12C* cor > 0.61, FDR < 2e-40).

## Discussion

Our study revealed extensive TE expression in tumors, which strongly associated with the expression of innate immune genes and triggered the production of polypeptides that are processed and presented on MHC-I molecules. These finding were made possible by the development of a novel computational approach that simultaneously quantified the expression of host genes and all annotated repetitive elements. Our extensive computational characterization demonstrated that the repetitive transcriptome, which had been previously ‘hidden’ to RNA-seq studies contained features strongly correlated to cancer cell phenotypes. These features are potentially cancer transcriptional fingerprints and may provide insights for future therapies.

Key findings included the demonstration of transcriptional activities from all five classes of TE, including DNA transposons, with an enrichment seen in the evolutionarily youngest TEs. Much of the TE expression was derived from intergenic loci, supporting autonomous expression of many TEs rather than read-through resulting from host, protein-coding genes. Advancing well-established observation of global demethylation in many cancer types, we discovered a much higher rate of DNA demethylation in regions of TE; *REdiscoverTE* permitted the demonstration that TE expression is tightly linked to proximal DNA demethylation in tumors. Using data from 7000 + patient tumors from TCGA, we provide the first the statistical demonstration that total TE expression accounts for substantial fractions of variance-explained for many gene expression signatures including type I interferon response, DNA damage repair response, cell cycle, and *P53* signaling. By expanding the reference cellular proteome and MHC class I peptidome to include TEs, we uncovered that TE transcripts can be translated and presented on the MHC-I molecules of tumor cells, with peptides stemming from intronic and intergenic HERV, LINE, SINE, and SVA loci. Tumor presentation of TE-derived MHC-I peptides represent an antigen space for T-cell recognition that is seldom explored. Consistent with a growing body of recent studies, these findings suggest TE expression in the tumor, whether spontaneous or induced under epigenetic therapy have important clinical implications for cancer immunotherapy^4–8,36,37,39,40^. Synergistic effect between epigenetic therapy and immunotherapy^38^ have been observed in preclinical models, where induction of antiviral immunity to retroviral expression is proposed to be a mechanism of tumor regression. In particular, for renal clear cell carcinoma (ccRCC or KIRC), where tumors have low mutational burden yet are highly immunogenic, HERV activity has been proposed to be a source of inflammation^6^ and recently associated with response to immunotherapy^8^. It remains to be seen, however, if epigenetic agents can be safely combined as a strategy to achieve improved efficacy in humans. Finally, T-cell populations recognizing the same HERV antigens have been identified in renal clear cell carcinoma tumors from multiple patients^8^, which offers evidence that TE antigens can be shared tumor antigens and should be explored as potential vaccine targets. In sum, our comprehensive in silico characterization of TE activities in tumors offers a number of predictions for experimental validation.

Regarding the computational methods presented herein, *REdiscoverTE* does not rely on consensus sequences, traditional short-read aligners, exclusion of multi-mapping reads, nor does it utilize step-wise operations that potentially introduce read-assignment bias^6,17,18,41,42^. In this study, we have focused on TE expression in cancer, however, the method automatically quantifies expression of all repeats including those that are not transposable, e.g., satellites, which have been implicated in epithelial tumors^43^. This method is broadly applicable to landscape profiling of the RE/TE expression in research areas beyond cancer, including autoimmune^44^, and neurodegenerative diseases^45,46^, as well as normal embryonic stem cell development where TE activation is a hallmark of cellular identity and pluripotency^42,47–50^. At last, *REdiscoverTE* can be adapted to apply to transcriptomic analysis of TEs and REs for any organisms whose genome contain such elements (Slotkin et al.^51,52^).

## Methods

### REdiscoverTE

*REdiscoverTE* uses the light weight-mapping method, *Salmon*^15^, for repetitive element expression quantification.

Generating *REdiscoverTE* reference transcriptome: Salmon version 0.8.2 was used to generate quasi mapping index. The reference transcriptome includes:

1. Distinct RNA transcript sequences (*n* = 98,029) from the GENCODE release 26 basic^13^
2. RepeatMasker elements (*n* = 5,099,056) from the standard chromosomes, excluding all polyA repetitive elements (Supplementary Fig. 1a, b). (Smit AFA, Hubley R, Green P. RepeatMasker Open-3.0. http://www.repeatmasker.org. 1996–2010)
3. Distinct sequences representing GENCODE RE-containing introns (*n* = 185,403) and excluding any regions overlapping with exons on either strand since we analyzed non-strand-specific RNA-seq.

Two transcriptome indices were built, one without (index 1) and the other with (index 2) the inclusion of RE-containing introns. We showed with simulation significant performance improvement by index 2 over index 1 (Supplementary Fig. 4e, f). As a result, *REdiscoverTE* transcriptome includes the RE-containing introns listed above.

*Salmon* version 0.8.2 was used to quantify RNA-seq data with adjustment for GC content bias and sequence specific bias options. *REdiscoverTE* reference transcriptome described above was used. *Salmon* produces quantification results in two ways: transcript-per-kilobase-million (TPM) and number of reads. We have chosen to use read counts for all downstream analysis.

Post-*Salmon* quantification, RE, and host gene transcripts were aggregated separately. Host isoforms were aggregated to the gene level according to *ensembl* gene ID. All aggregation and downstream analysis of the aggregated expression were performed using R^53^. R: A language and environment for statistical computing. R Foundation for Statistical Computing, Vienna, Austria. URL https://www.R-project.org.).

Aggregation of RE expression to the subfamily level: owing to the high degree of sequence homology among numerous copies of REs from the same subfamily, quantification at the individual locus level is highly susceptible to noise. Therefore, *Salmon* quantification of read counts for individual REs were aggregated to the level of RE subfamily, family and class according to hierarchies defined by the human Repeatmasker for Hg38 (a.k.a., repName, repFamily, repClass, respectively) (Supplementary Fig. 1a). In this summing process, RE expression was further distinguished by the genomic locations of individual REs with respect to genes. The Gencode annotation of human transcriptome^13^ Version 26 Basic (https://www.gencodegenes.org) was used to define REs’ relationship with respect to host genes. Gencode GTF/GFF file defines the following basic categories of features: gene, transcript, coding exon (CDS), exon, UTR. We inferred intronic and intergenic regions from these features (Supplementary Fig. 1b pie chart), then simplified these categories of features into three mutually exclusive regions: exons (union of all exons and UTRs), introns (union of intronic regions excluding any overlap with exons), and intergenic regions. The R package *GenomicRanges*^54^ was used to perform range overlap operations. For REs overlapping multiple contexts (e.g., a RE that resides at an exon-intron boundary), their locations are assigned with the following priority: exon > intron > intergenic. For example, a RE residing at the exon-intron boundary is considered as an exonic RE.

### *REdiscoverTE* performance benchmarking with RSEM simulation

To benchmark the accuracy of *REdiscoverTE*, we carried out RSEM^16^ simulations to create fastq files with ground truth on expression levels (TPM) of all features in the transcriptome. To create realistic gene and RE expression levels we first used RSEM to learn sequence statistics from two TCGA RNA-Seq samples with different proportions of RE reads estimated by REdiscoverTE: one with 5.4% of reads derived from REs as estimated by *REdiscoverTE* (THCA, normal sample, TCGA-EL-A3ZS-11A-11R-A23N-07), and one with 11.5% of reads derived from REs (LAML, tumor sample, TCGA-AB-2955-03A-01T-0734-13), then generated corresponding simulated fastq files based on learned models (Supplementary Fig. 1c step 2, step 3) and two additional modifications described below.

A main goal of the simulation is to evaluate *Salmon*’s ability to quantify gene expression stemming from highly repetitive and similar features. We considered the added complexity where some REs can overlap with host gene features that are also expressing mRNAs, e.g., host gene transcripts or retained introns. The following two modifications were made to the default RSEM simulation process to address these issues (also described in Supplementary Fig. 1c):

1. To evaluate Salmon’s ability to distinguish RE expression from overlapping host transcripts (Fig. 1a orange reads from RE #2 vs. blue reads from exon), we simulated RE expression at varying levels above the host gene expression. We chose to focus our simulations on those RE subfamilies that have copies residing in all three types of genomic regions with respect to genes: exonic, intronic, and intergenic regions. Out of 15,440 RE subfamilies, there are 3659 subfamilies contain at least one copy of RE in each of exonic, intronic, and intergenic regions. After exluding simples repeats, there were 1135 subfamilies that satisfy this criterion (Supplementary Fig. 1c Venn Diagram). We randomly chose 1000 non-Simple Repeat subfamilies from these 3659 to evaluate with simulation experiments (Supplementary Fig. 1c workflow). If an RE overlapped with multiple isoforms or genes, for simplicity, we randomly chose one isoform to simulate for every RE residing within the transcript. In total, 1,969,915 REs from 1000 non-Simple Repeat subfamilies were simulated; 63,021 of them overlapped with genes.
2. To evaluate *Salmon*’s ability to distinguish RE expression from retained introns (Fig. 1a orange reads from RE #3 vs. green reads from retained intron), two isoforms were simulated for genes with intronic REs: one with the RE-containing intron retained, and one without the intron.

After *RSEM* learned statistical profiles from the two TCGA fastq files, and before generating simulated fastq files, we manually changed the TPM values in the *isoforms.results* output file from rsem-calculate-expression in order to generate more variation in intron retention levels and RE to RE-containing gene expression level ratio. Supplementary Fig. 1d provides the final profiles of these simulations.

### Compare *REdiscoverTE* with three existing methods

*RepEnrich*^17^ is a two-step repetitive elements quantification method: step 1–alignment of RNA-seq reads to hg38 using Bowtie^55^, step 2–applying *RepEnrich* script to reads uniquely mapped to repeatmasked regions and multi-mapped reads from step 1 using *RepEnrich* pre-defined repetitive pseudogenomes as reference. *RepEnrich* pseudogenomes are defined for 1000 + RE subfamilies (excluding simple repeats and low complexity repeats), each is a concatenation of all repetitive elements in the subfamily with additional flanking sequences and spacers.

We benchmarked performance of *REdiscoverTE* against *RepEnrich* on RSEM simulated RNA-seq data. We followed default workflow of *RepEnrich*. Performance of *REdiscoverTE* and *RepEnrich* were evaluated using the metric MARD at the level of subfamily, where MARD is defined as in *Salmon* publication:^15^

$${\mathrm{MARD}} = \frac{1}{N}\mathop {\sum }\limits_{i = 1}^N \frac{{|{\mathrm{Salmon}}\;{\mathrm{count}}_i - {\mathrm{Simulated}}\;{\mathrm{count}}_i|}}{{{\mathrm{Salmon}}\;{\mathrm{count}}_i + {\mathrm{Simulated}}\;{\mathrm{count}}_i}}$$

Here *N* is the total number of features, where features could be individual RE transcripts or aggregated features such as RE subfamily.

To directly compare the ERV quantification results from ref. ^6^, we created a *Salmon* reference transcriptome that included 90 k human transcripts and the same 124 sequences for the 66 ERVs analyzed by the authors (from ref. ^56^). RNA-seq from 5217 TCGA samples (20 cancer types) were quantified by *Salmon* (Version 0.6.0). ERV read counts were normalized by total counts mapped to genes, similar to Rooney et al. where ERV expression was normalized by total counts of reads mapped to genes.

To compare with *SalmonTE*^18^, *SalmonTE* v0.4 was downloaded from https://github.com/LiuzLab/SalmonTE.

### *REdiscoverTE* quantification of TE expression

TruSeq adapters were trimmed by Cutadapt (http://cutadapt.readthedocs.io/en/stable/) from both TCGA and CGP. *REdiscoverTE* was run as described above for whole-transcriptome expression quantification.

Following expression aggregation: isoform level to gene level, individual REs to RE subfamilies, two expression count matrices were created for each data set, one for gene expression, the other for RE expression. We chose to calibrate both expression matrices using total counts of gene expression, which we considered to be more stable across samples. Expression normalization was performed in R using the Bioconductor packages *edgeR*^20^ using “RLE” method by function *calcNormFactors*. log2CPM is then calculated with prior count set to 5. After normalization, for the RE expression matrix, we focused on the 1052 TE subfamilies for downstream analysis in this study.

To control for potential batch effect and patient-to-patient variation, only tumor and matched adjacent normal samples are used for differential expression analysis. Cancer types with fewer than 10 normal samples were excluded from this analysis; 13 TCGA cancer types and five CGP cancer types satisfied this threshold. The R packages *limma* and *voom*^21,22^ were used for differential expression analysis using aggregated count matrices as input. Prior to differential expression analysis, two filters were applied to exclude genes or TEs with low expression, requiring (1) at least 10% of samples that have counts greater than zero, and (2) a log2(CPM) threshold. The log2(CPM) threshold was determined independently for each cancer type based on visual inspection of the mean-variance trend (estimated by the *voom* function in limma) to ensure variance was monotonically decreasing for low mean expression. Within each cancer type, raw *p* values resulting from differential expression (*t* tests on log2 fold change was different from 0) are adjusted by Benjamini–Hochberg (BH) approach to control false discovery rate (FDR)^57^. Differentially expressed genes/TEs were determined at the threshold of: abs(log2 fold change) > 1 and FDR < 0.05.

### DNA methylation analysis

TCGA Illumina 450k Infinium methylation arrays were processed using the “lumi”^58^. Raw array data were background corrected (lumiB method) and variance stabilized and normalized (lumiT and lumiN methods). Beta values were calculated per CpG site by flooring intensity values at zero and then calculating

$${\mathrm{Beta}} = \frac{{{\mathrm{methylated}}\;{\mathrm{density}}}}{{{\mathrm{methylated}}\;{\mathrm{density}} + {\mathrm{unmethylated}}\;{\mathrm{density}} + {\mathrm{alpha}}}}$$

where alpha is a regularization parameter set at the default of 100 recommended

by Illumina^59^. *M* values were transformed from Beta values by:

$$M = {\mathrm{log}}2\left( {\frac{{{\mathrm{Beta}}}}{{1 - {\mathrm{Beta}}}}} \right)$$

Liftover of CpG sites in 450 K array to hg38: Hg19 annotation of 450 K probes was obtained using R package “IlluminaHumanMethylation450kanno.ilmn12.hg19”^60^. IlluminaHumanMethylation450kanno.ilmn12.hg19: Annotation for Illumina’s 450 K methylation arrays. R package version 0.6.0.) Using the liftOver utility provided by UCSC genome browser, physical coordinates of 450 K probes in hg19 annotation were lifted to hg38 reference genome. 485,441 out of 485,512 CpGs were successfully converted to Hg38, 71 failed owing to position removal in Hg38 assembly.

In order to identify differentially methylated cytosines (DMCs), *M* values of DNA methylation data were used to fit the linear regression model with tumor/normal status and patient ID as covariate using *lmFit* from the R package *limma*^21^. To control for potential batch effect and patient-to-patient variation, only tumors (*N* = 237) with matched normal (*N* = 236) samples were included for analysis in 10 TCGA cancer types (Supplementary Table 3) that had both DNA methylation and RNA-seq data.

DMCs were defined as CpGs with the average absolute beta value change (Δbeta) ≥ 10% and FDR < 0.05. DMCs are called demethylated if the average beta value in tumor is lower than normal samples, and methylated if the average beta value in tumor is higher than normal.

Δbeta value for each CpG site is defined for tumor and matched normal sample pairs as:

$$\Delta {\mathrm{beta}} = {\mathrm{beta}}_{{\mathrm{tumor}}}-{\mathrm{beta}}_{{\mathrm{normal}}}$$

Distribution of 450 K CpGs in different genomic features

Similar to RE expression analysis, CpGs were classified into exonic, intronic and intergenic CpGs according to the genomic features defined above. The genomic distribution of all 450 K CpGs and CpGs overlapping with TEs were visualized in Supplementary Fig. 3a. In total, there were 70,004 CpGs located within TEs that overlapped with 59,739 individual elements from 992 TE subfamilies.

Spatial profile of methylation around TEs were analyzed by extracting CpG sites near intergenic TEs. CpGs located outside but nearby individual intergenic TE elements were binned into two categories for further analysis: those within 1 kb of TEs and those within 10 kb of TEs. Owing to the lack of functional annotation for all TE transcripts, the most 5′ bp of each individual TE as annotated by *RepeatMasker* was used as proxy for transcription start site (TSS).

For the 1 kb methylation spatial analysis, all 450 K Array CpG sites within 500 bp ± of TSS for each intergenic TE were extracted using the *findOverlaps* function in the R package *GenomicRanges* with strand information taken into account. This resulted in 90,950 TE-proximal CpG sites for 1007 TE subfamilies.

For these 1007 TE subfamilies, CpG sites from a bigger, 10 kb window: 5 kb up- and downstream from the start and end coordinates of individual intergenic TEs, were extracted, resulting in 155,360 CpG sites.

For spatial profile analysis (e.g., Figure 3f, g) CpGs within TEs were represented using a proportional distance as follows: TEs from the same subfamily were length normalized to create proportional position within the TE, ranging from 0 to 100% that correspond to the start and end of TE.

### Correlation between DNA methylation and TE expression

Two types of correlation analyses were carried out, both using Pearson correlation on aggregated intergenic TE expression at the subfamily level and *M* values of CpG sites, chosen over beta to obtain higher statistical power. The first one uses the per-sample average *M* values at all CpGs within 1 kb of TEs (e.g., Figure 3e, Supplementary Fig. 3f column 3) the second one is performed at each CpG site around 5 kb ± of all intergenic elements in the TE subfamily, using *M* values from all samples at the given CpG site (e.g., Figure 3f, Supplementary Fig. 3f columns 4 and 5). FDR was obtained by adjusting *p* values for multiple testing (Benjamini & Hochberg) across the 1007 tests within each cancer type. Significant correlation was defined as FDR < 0.05 and |cor| ≥ 0.4.

TE demethylation enrichment score. We defined methylation state as the ratio of number of demethylated vs. number of over-methylated DMC sites. Methylation state is 1 when there are equal number of demethylated and over-methylated DMCs, >1 when there is bias in the direction of demethylation, <1 when there is bias toward over-methylation.

We then computed a TE demethylation enrichment score (Supplementary Table 3) as the ratio of within-TE methylation state (using DMC CpG sites in intergenic TEs) to global methylation state (using all DMC sites). This enrichment score is 1 when the methylation state in TE is comparable to that of the global methylation state, > 1 when a higher proportion of TE DMCs are demethylated, <1 when a smaller proportion of TE DMCs are demethylated.

$${\mathrm{TE}}\;{\mathrm{demethylation}}\;{\mathrm{enrichment}} = \frac{{{\mathrm{N}}_{{\mathrm{demethylated}}\;{\mathrm{DMC}}\;{\mathrm{in}}\;{\mathrm{TE}}}/N_{{\mathrm{over}} - {\mathrm{methylated}}\;{\mathrm{DMC}}\;{\mathrm{in}}\;{\mathrm{TE}}}}}{{{\mathrm{N}}_{{\mathrm{demethylated}}\;{\mathrm{DMC}}\;{\mathrm{anywhere}}}/N_{{\mathrm{over}} - {\mathrm{methylated}}\;{\mathrm{DMC}}\;{\mathrm{anywhere}}}}}$$

### Association between gene signatures and TE expression

Twenty-four gene signatures associated with major cellular pathways related to cancer, DDR and immune response were selected from previous publications (Supplementary Data 4). The R package *multiGSEA* (https://github.com/lianos/multiGSEA) was used to score gene signature expression based on singular value decomposition.

In order to estimate the immune content within tumor samples, we applied *xCell*^32^, a recently developed gene signature-based approach for tissue cellularity de-convolution within RNA-seq data, to TCGA samples and obtained the cellularity enrichment scores for 64 cell types, including lymphoid and myeloid cell types (Supplementary Fig. 4c). We further confirmed the accuracy of *xCell* estimations by examining the concordance between certain cell types (e.g., CD8 + T cells) and related gene signature scores (e.g., CD8 + effector T cells) computed with *multiGSEA* (Supplementary Fig. 4c). In addition, for each sample, we defined *total lymphoid content* as the sum of 21 lymphoid cell scores: CD8 + T cells, NK cells, CD4 + naive T cells, B-cells, CD4 + T cells, CD8 + Tem, Tregs, plasma cells, CD4 + Tcm, CD4 + Tem, memory B-cells, CD8 + Tcm, naive B-cells, CD4 + memory T cells, pro B-cells, class- switched memory B-cells, Th2 cells, Th1 cells, CD8 + naive T cells, NKT and Tgd cells. *Total myeloid content* was defined as the sum of 13 cell scores: monocytes, macrophages, DC, neutrophils, eosinophils, macrophages M1, macrophages M2, aDC, basophils, cDC, pDC, iDC, mast cells.

For each cancer type, Spearman correlation coefficients between log2CPM expression of 1052 TE subfamilies and 24 gene signature scores were computed using the R package for partial correlations *ppcor* (https://CRAN.R-project.org/package=ppcor), with tumor purity as a covariate. Benjamini–Hochberg approach was used to control FDR within each cancer type separately.

In addition, Spearman correlation coefficients between 1052 TEs and 64 *xCell* scores were computed using the same method.

### Lasso associations between gene signature and TE expression

To identify top TE subfamilies associated with each of the 24 cellular pathways and gene signatures, we exploited Lasso regularized regression–generalized linear model via penalized maximum likelihood using the R package *glmnet*^31^. In order to account for variations of cellular content that existed between tumor samples, we included tumor purity as well as abundance of total lymphoid and myeloid content (*xCell* section above) as parameters in the lasso model. To avoid any possible bias introduced by normal-tumor status, only tumor samples were used in the regression model.

Three Lasso models were implemented for each gene expression signature (*N* = 24) and each cancer type (*N* = 25):

Model (1): Cellularity-only

$${\mathrm{Signatures}}\sim {\mathrm{tumor}}\;{\mathrm{purity}} + {\mathrm{lymphoid}} + {\mathrm{myeloid}}$$

Model (2): Cellularity + true TE data:

$${\mathrm{Signatures}}\sim {\mathrm{true}}\;{\mathrm{TE}}\;{\mathrm{data}} + {\mathrm{tumor}}\;{\mathrm{purity}} + {\mathrm{lymphoid}} + {\mathrm{myeloid}}$$

where true TE data is a Nx1052 matrix containing 1052 TE expression in units of log2CPM from *N* samples.

Model (3): Cellularity + permuted TE data

$${\mathrm{Signatures}}\sim {\mathrm{permuted}}\;{\mathrm{TEs}} + {\mathrm{tumor}}\;{\mathrm{content}} + {\mathrm{lymphoid}} + {\mathrm{myeloid}}$$

where permuted TE data is a row-wise scrambled version of the true TE data.

Ten rounds of tenfold cross-validation was performed for each regression. Lasso coefficients at one standard error of the minimum mean cross-validation errors (lamda 1SE) were used. Each Lasso fit yielded a sparse set of predictors—variables with non-zero coefficients, corresponding to TE subfamilies with significant contributions to the variability of a given gene signature. We then ranked all 1055 dependent variables (TEs and three cellularity scores) by their average absolute coefficient values across cancer types to select the top 20 predictors associated with the gene signature of interest. To produce the Lasso rank coefficient heatmap (Supplementary Fig. 4d), we indicated the rank of these top predictors by their absolute coefficient values within each cancer type. Dots corresponds to a coefficient of zero for a given cancer type (also shown in Supplementary Data 5).

Post Lasso regression, deviance ratio from the models (fraction of deviance explained) were averaged across the 10 rounds of cross-validation and used as *R*^2^ values for these models.

### TE peptide identification

MS raw data files for the global proteome (unenriched peptides) and MHC-bound peptidome (pan-MHC-I enriched peptides) were obtained from PRIDE (PXD003790) and SysteMHCAtals (SYSMHC00007), respectively.

To enable identification of TE-derived peptides in the GBM proteome and MHC-bound peptidome data, we collected nucleotide sequences at all individual loci for the 62 TE subfamilies that were significantly overexpressed at either the intergenic or intronic regions upon 5′aza-treated condition, performed six frame translations (both forward and reverse direction), then fragmented the resulting amino-acid sequences at all stop codons. This yielded ~ 1.1 M peptide fragments, ranging 7–1321 amino acids in length. The peptide fragments were combined with the human protein sequences in Uniprot (downloaded Jan 1, 2017) and common contaminant proteins to create a database used for searching non-MHC-enriched MS data. TE-derived peptide fragments were further reduced into 4.6 M 11-mers generated with a moving window of eight amino-acid overlaps, with duplicates removed. This 11-mer database was also combined with the human protein sequences in Uniprot and common contaminant to create a database used for searching MHC-enriched MS data.

Raw MS data were analyzed using PEAKS Studio (Bioinformatics Solutions Inc., v8.5)^61^. In brief, raw MS data were refined and sequence tags were identified by a de novo search algorithm. Identified sequence tags were used in the assignment of peptide sequences to MS data through a database search. For all database searches, the following parameters were used: precursor tolerance = 25 ppm, fragment ion tolerance = 0.02 Da, enzyme = none, variable modifications include deamidation [N or Q, 0.98 Da] and oxidation [M, 15.99 Da], and max number of variable mods = 3. Data were filtered to 1% FDR at the peptide level, but owing to TE peptide fragments being represented as multiple “protein” entries within the database protein level FDR was not performed. For MHC-bound peptidome data a median of three peptide spectral matches (PSMs) were identified for non-TE peptides, owing to this TE peptides were considered high confidence if they had been identified in ≥ 3 spectra.

In addition, we performed an identical search against a database that did not include the TE peptide 11-mers in order to determine whether TE PSMs mapped to alternative sequences (Supplementary Data 9). A total of 555 PSMs mapped to TE peptides when the 11-mer peptides were included in the database. Of these, 487 failed to match a peptide sequence at 1% FDR when TE peptide 11-mers were excluded from the database. Of the remaining 68 PSMs, 64 matched to Trembl, Uniprot entries, which are short RNA transcript reads that likely originate from expressed TE peptides. The remaining four spectra matched to alternative proteins in Uniprot (3) or a decoy protein entry (1). If we consider these four spectra false observations we can estimate our experimental FDR to be ~ 1.4%.

For further validation of TE peptide identification, we synthesized 15 of the 83 unique peptides and analyzed them by MS. All MS analyses were performed on an Orbitrap Fusion MS (ThermoFisher Scientific, San Jose, CA) with peptides separated over a nano-LC column (100 µm I.D. packed to 25 cm with Waters M-Class BEH 1.7 µm packing material) by a gradient delivered by a Waters NanoAqcuity nano-LC. For each synthetic TE peptide, 250 fmol was injected and analyzed by MS utilizing various collision energies (HCD at 20, 25, 30, and 35 NCE) in order to match fragmentation spectra of Shraibman et al. Synthetic peptide MS data were analyzed in PEAKS in an identical manner to the MHC-bound peptidome data. Annotated spectra for the synthetic and experimental spectra were manually compared with validate peptide identifications. Through this process we were able to confirm 17 of 18 peptide spectra as visual matches, adding further confidence to TE peptide identifications.

### MHC class I peptide exchange

Recombinant HLA-A*03:01 MHC-I was refolded in the presence of a conditional peptide ligand that contains a UV-sensitive amino acid, as previously described^62^. The resultant purified, stable complex was incubated in the presence of 100-fold molar excess of synthetic TE-derived peptides of interest. HLA-A*03:01 was present at a concentration of 50 µg/ml (1.04 µm) in 25 mm TRIS pH 8.0, 150 mm NaCl, 2 mm EDTA, 5% DMSO. The peptide exchange reaction mixture was incubated for 25 min under a UV lamp set to 365 nm to induce cleavage of the UV-sensitive amino-acid 3-amino-3-(2-nitro)phenyl-propionic acid. Samples were then incubated at room temperature, overnight, to allow for peptide exchange to occur. Upon cleavage of the conditional peptide ligand, synthetic TE-derived peptides with suitable properties (affinity, solubility) exchanged into the complex displacing any fragments of the cleaved conditional ligand.

### 2D- LCMS characterization of peptide exchange

To determine successful exchange of TE-derived peptides into HLA-A*03:01 complexes, a 2-dimensional liquid chromatography MS method was used. The first dimension LC method employed an analytical SEC column (Agilent AdvanceBio SEC 300 Å, 2.7 µm, 4.6 × 15 mm) to separate intact complex from excess peptide run at an isocratic flow of 0.7 ml/min in 25 mm TRIS pH 8.0, 150 mm NaCl for 10 min. A sampling valve collected the entirety of the complex peak that eluted between 1.90 and 2.13 min in a volume of 160 µl and injected it onto the second dimension reversed phase column (Agilent PLRP-S 1000 Å, 8 µm, 50 × 2.1 mm). The second dimension column was exposed to a gradient of 5–50% mobile phase B in 4.7 min at 0.55 ml/min with the column heated to 80 °C. Mobile phase A was 0.05% TFA. Mobile phase B was 0.05% TFA in acetonitrile. The column eluent was sent to an Agilent 6224 TOF LCMS for MS data acquisition.

HLA-A*03:01 complex peak area (detected at 280 nm) in the first dimension and mass spec detection of the peptide in the second dimension are used to determine successful exchange. Successful exchange of a peptide into the complex after cleavage of the conditional ligand during the peptide exchange reaction stabilizes the complex and results in nearly complete recovery of the starting complex measured in the first dimension SEC analysis. The peptide that has exchanged into the complex can then be detected in the second dimension, where the complex is run under denaturing conditions with mass spectral analysis allowing for direct detection of the peptide of interest. Unsuccessful peptide exchange reactions result in destabilized complex after the cleavage of the conditional ligand when a peptide fails to bind to and stabilize the complex. This is measured as a reduction in A280 peak area of the complex on SEC and an absence of peptide in the second dimension. In some cases, such as for peptide RLAPRPASR, no reduction in peak area was observed, however the peptide was not detected by MS. A small number of peptides, due to their properties, are not captured by the second dimension chromatography column and method. In these cases, the peak area recovery is enough to suggest successful exchange when the proper experimental controls are used.

### Reporting summary

Further information on research design is available in the Nature Research Reporting Summary linked to this article.

## Supplementary information

Supplementary Information

Description of Additional Supplementary Files

Supplementary Data 1

Supplementary Data 2

Supplementary Data 3

Supplementary Data 4

Supplementary Data 5

Supplementary Data 6

Supplementary Data 7

Supplementary Data 8

Supplementary Data 9

Reporting Summary

## Data Availability

TCGA methylation and RNA sequencing data sets for all cancer types may be downloaded from the TCGA data portal [https://portal.gdc.cancer.gov/]. Tumor purity values were downloaded from NIH/NCI GDC PanCanAtlas Publications website [https://gdc.cancer.gov/about-data/publications/pancanatlas]. CGP RNA sequencing data have been deposited in the European genome-phenome archive under the following accession codes: small cell lung cancer screen, EGAS00001000334; colon cancer screen, EGAS00001000288; Exome sequencing, RNA Sequencing, SNP array profiling of gastric tumor samples and cell lines, EGAS00001000736 and non-clear cell renal cell carcinoma, EGAS00001000926. Matched RNA sequencing, proteome and MHC-I peptidome data previously published in^38^ on three glioblastoma cell lines before and after decitabine treatment were downloaded from PRIDE under the accession code PXD003790 and GEO under the accession code GSE80137. All the other data supporting the findings of this study are available within the article and its supplementary information files and from the corresponding author upon reasonable request. A reporting summary for this article is available as a Supplementary Information file.

## Code availability

All statistical analyses described in the manuscript were performed using custom software developed in R. Both the REdiscoverTE software and the REdiscoverTEdataR package which contains data and scripts used to make the main figures are available for download at http://research-pub.gene.com/REdiscoverTEpaper/.
