# Supplementary material for: Transposable element expression in tumors is associated with immune infiltration and increased antigenicity
Source: Nat Commun. 2019 Nov 19;10:5228. doi: 10.1038/s41467-019-13035-2 (PMC6864081; doi:10.1038/s41467-019-13035-2)
Supplement: Supplementary file 1 — Supplementary Information [file 41467_2019_13035_MOESM1_ESM.pdf]

Supplementary Information

TRANSPOSABLE ELEMENT EXPRESSION IN TUMORS IS ASSOCIATED WITH IMMUNE INFILTRATION AND INCREASED ANTIGENICITY

Kong et al.

Supplementary Figures

Supplementary Figure 1 (Related to Figure 1)

a

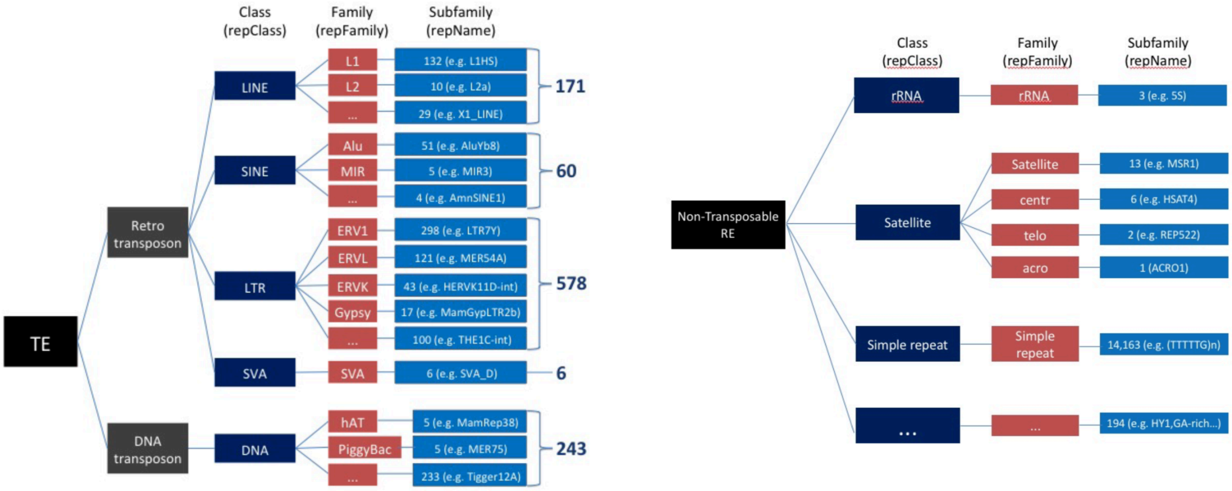

b

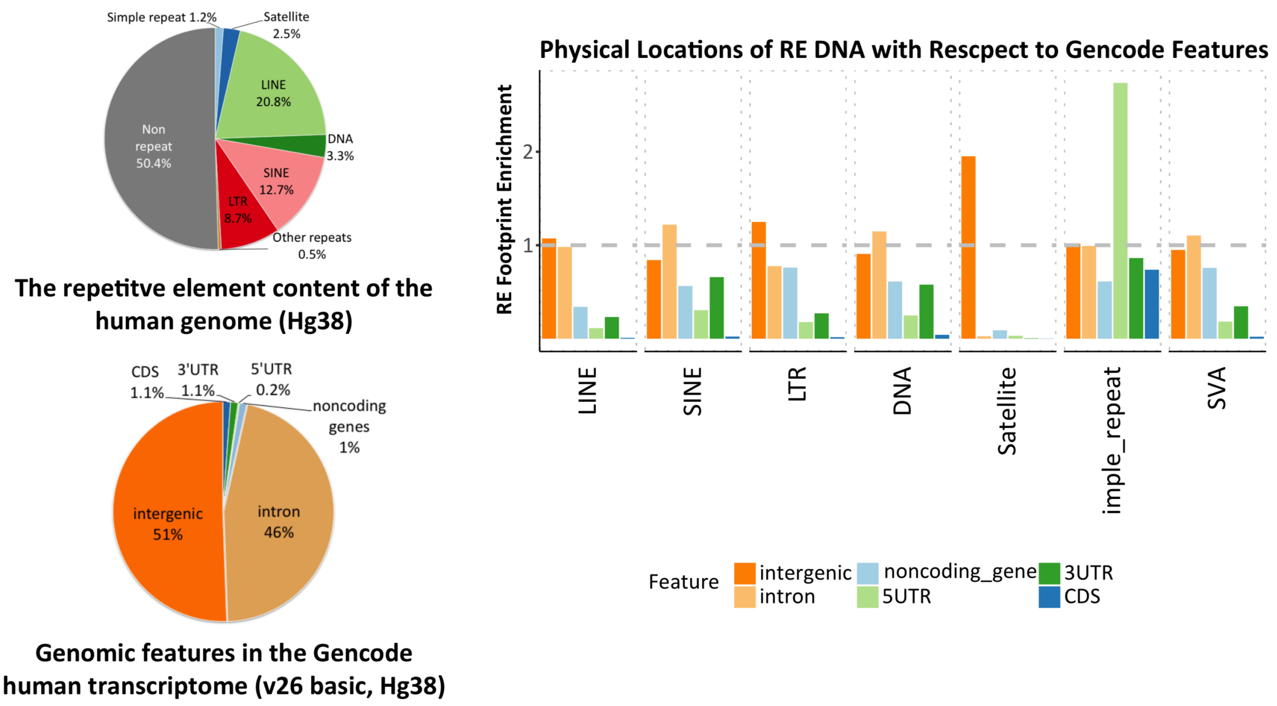
Simple repeat 1.2%
Satellite 2.5%
LINE 20.8%
DNA 3.3%
SINE 12.7%
LTR 8.7%
Other repeats 0.5%
Non repeat 50.4%
The repetitve element content of the human genome (Hg38)
CDS 1.1%
3'UTR 1.1%
5'UTR 0.2%
noncoding genes 1%
intergenic 51%
intron 46%
Genomic features in the Gencode human transcriptome (v26 basic, Hg38)
Physical Locations of RE DNA with Rescpect to Gencode Features
RE Footprint Enrichment
2
1
LINE
SINE
LTR
DNA
Satellite
imple_repeat
SVA
Feature
intergenic
intron
noncoding_gene
5UTR
3UTR
CDS

c

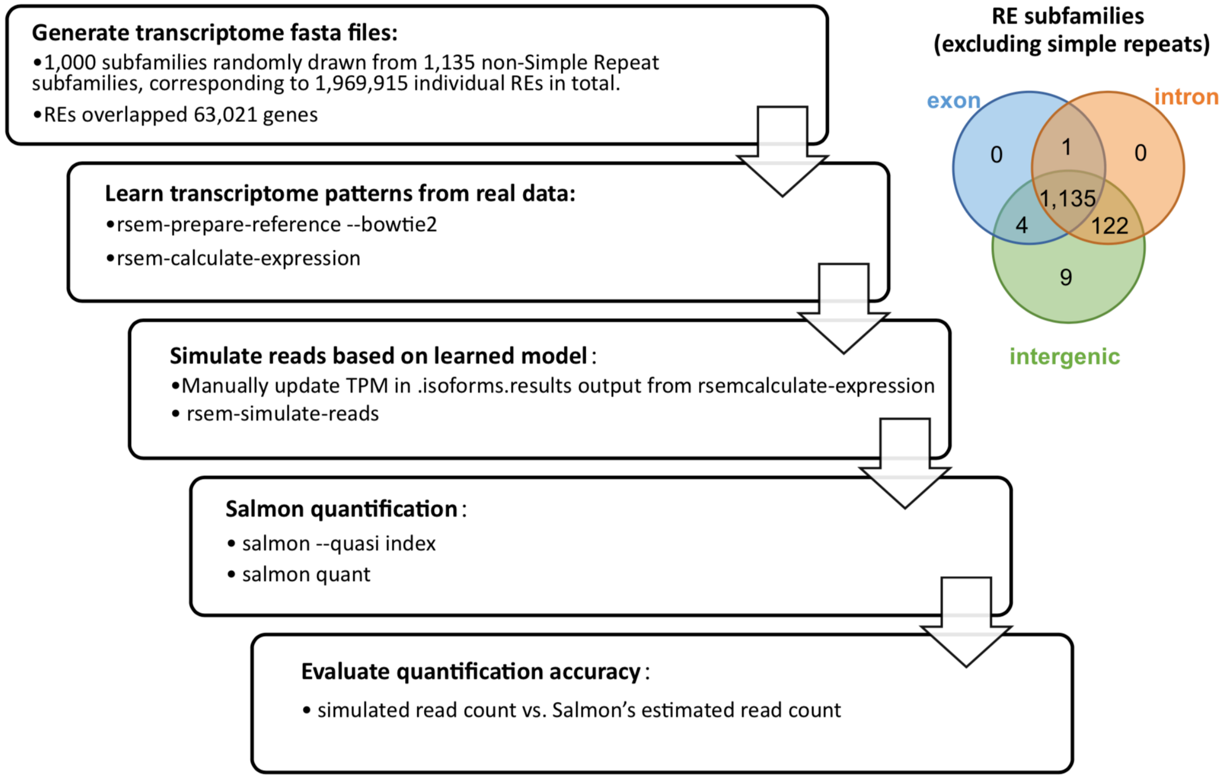
Generate transcriptome fasta files:
•1,000 subfamilies randomly drawn from 1,135 non-Simple Repeat subfamilies, corresponding to 1,969,915 individual REs in total.
•REs overlapped 63,021 genes
Learn transcriptome patterns from real data:
•rsem-prepare-reference --bowtie2
•rsem-calculate-expression
Simulate reads based on learned model :
•Manually update TPM in .isoforms.results output from rsemcalculate-expression
• rsem-simulate-reads
Salmon quantification :
• salmon --quasi index
• salmon quant
Evaluate quantification accuracy :
• simulated read count vs. Salmon's estimated read count
RE subfamilies (excluding simple repeats)
exon
intron
intergenic
0
1
0
1,135
4
122
9

d

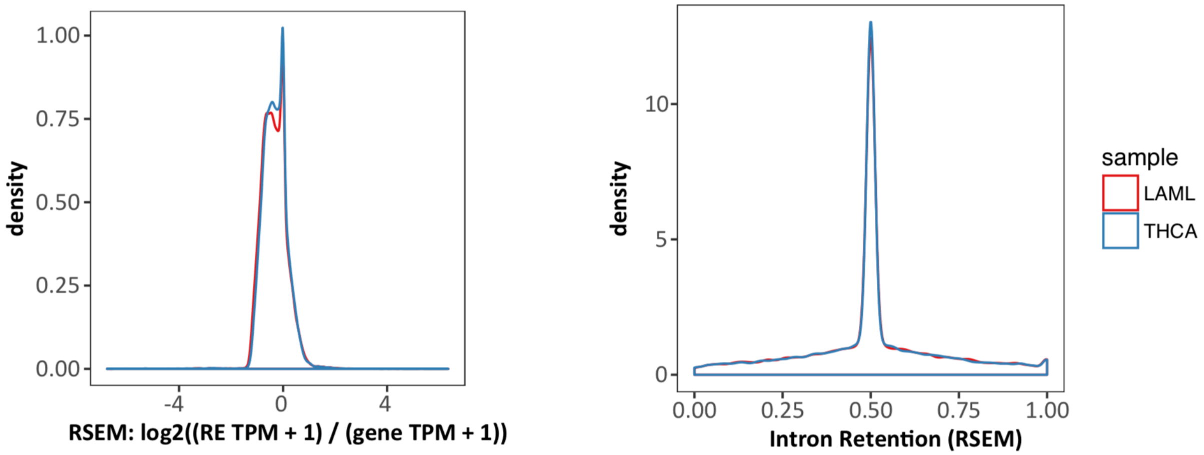
density
1.00
0.75
0.50
0.25
0.00
-4
0
4
RSEM: log2((RE TPM + 1) / (gene TPM + 1))
density
10
5
0
0.00
0.25
0.50
0.75
1.00
Intron Retention (RSEM)
sample
LAML
THCA

e

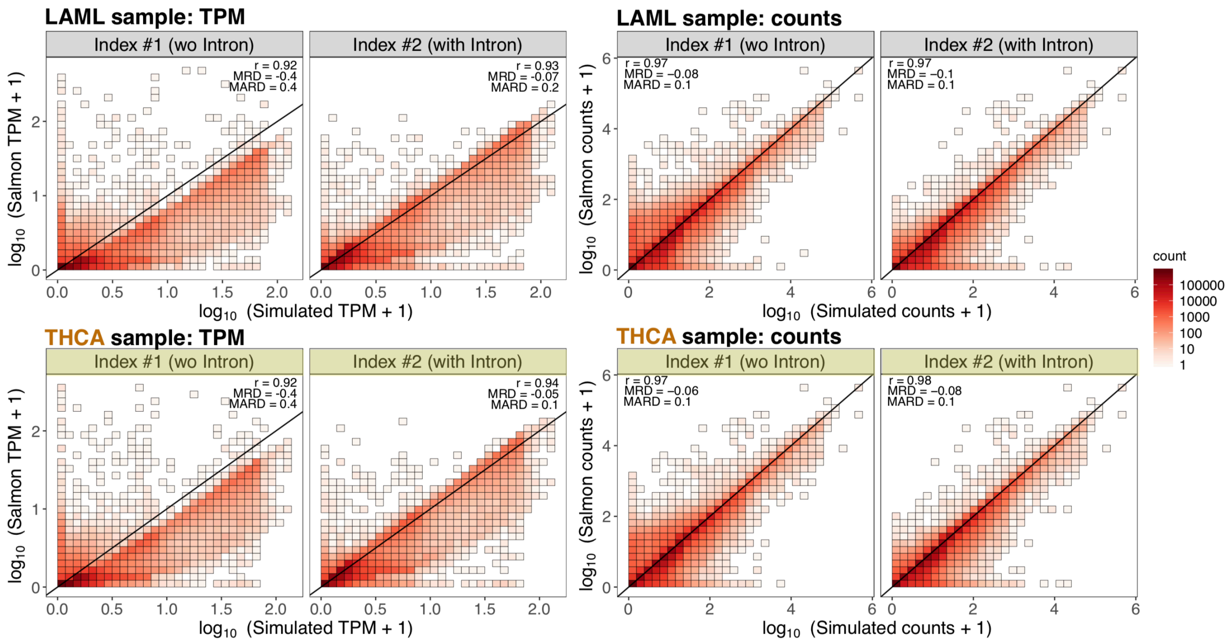
LAML sample: TPM
Index #1 (wo Intron)
Index #2 (with Intron)
r = 0.92
MRD = -0.4
MARD = 0.4
r = 0.93
MRD = -0.07
MARD = 0.2
log10 (Salmon TPM + 1)
log10 (Simulated TPM + 1)
LAML sample: counts
Index #1 (wo Intron)
Index #2 (with Intron)
r = 0.97
MRD = −0.08
MARD = 0.1
r = 0.97
MRD = −0.1
MARD = 0.1
log10 (Salmon counts + 1)
log10 (Simulated counts + 1)
count
100000
10000
1000
100
10
1
THCA sample: TPM
Index #1 (wo Intron)
Index #2 (with Intron)
r = 0.92
MRD = -0.4
MARD = 0.4
r = 0.94
MRD = -0.05
MARD = 0.1
log10 (Salmon TPM + 1)
log10 (Simulated TPM + 1)
THCA sample: counts
Index #1 (wo Intron)
Index #2 (with Intron)
r = 0.97
MRD = −0.06
MARD = 0.1
r = 0.98
MRD = −0.08
MARD = 0.1
log10 (Salmon counts + 1)
log10 (Simulated counts + 1)

f

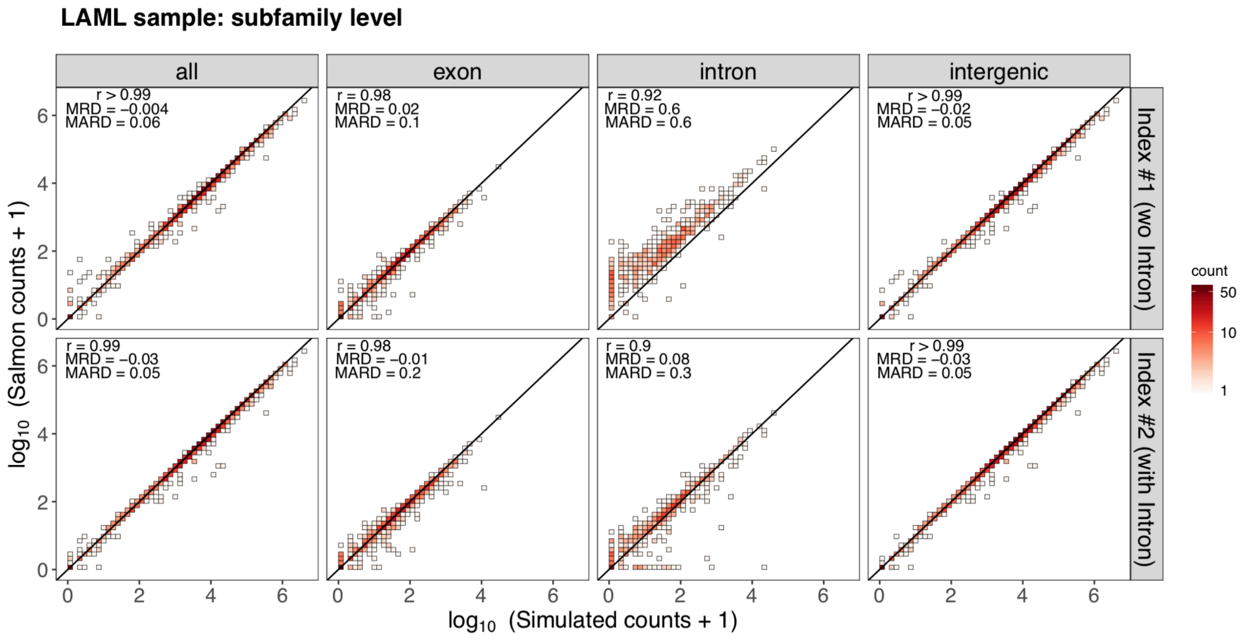
LAML sample: subfamily level
all
exon
intron
intergenic
Index #1 (wo Intron)
Index #2 (with Intron)
r > 0.99
MRD = −0.004
MARD = 0.06
r = 0.98
MRD = 0.02
MARD = 0.1
r = 0.92
MRD = 0.6
MARD = 0.6
r > 0.99
MRD = −0.02
MARD = 0.05
r = 0.99
MRD = −0.03
MARD = 0.05
r = 0.98
MRD = −0.01
MARD = 0.2
r = 0.9
MRD = 0.08
MARD = 0.3
r > 0.99
MRD = −0.03
MARD = 0.05
log10 (Salmon counts + 1)
log10 (Simulated counts + 1)
count
50
10
1

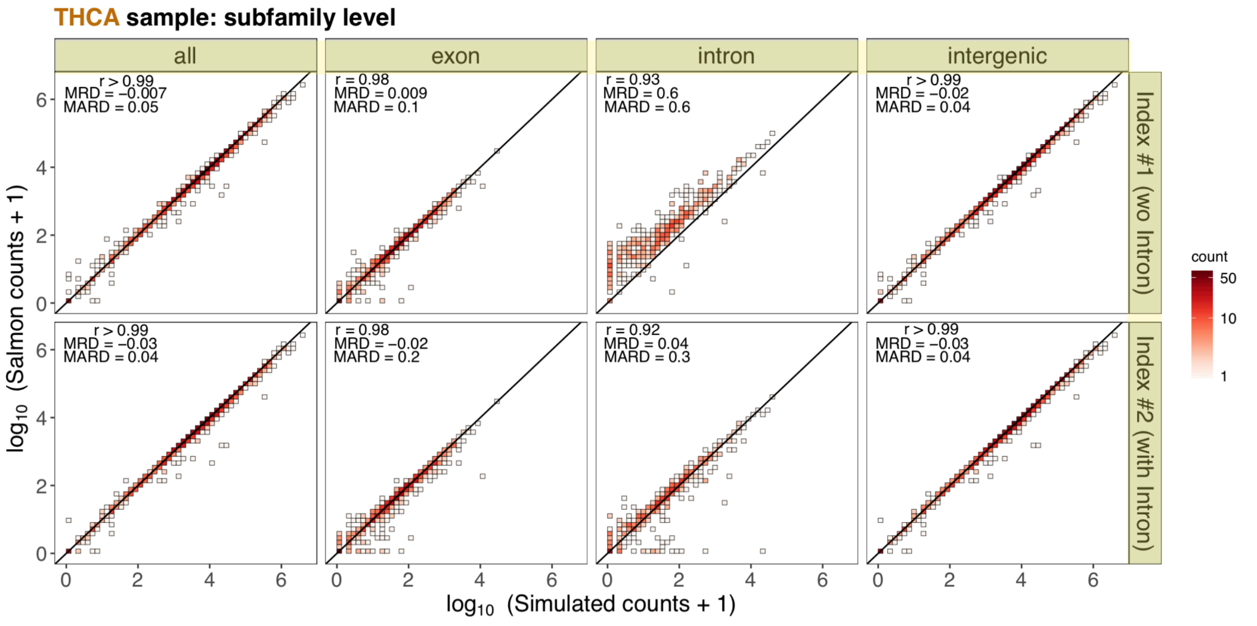
THCA sample: subfamily level
all
exon
intron
intergenic
Index #1 (wo Intron)
Index #2 (with Intron)
r > 0.99
MRD = −0.007
MARD = 0.05
r = 0.98
MRD = 0.009
MARD = 0.1
r = 0.93
MRD = 0.6
MARD = 0.6
r > 0.99
MRD = −0.02
MARD = 0.04
r > 0.99
MRD = −0.03
MARD = 0.04
r = 0.98
MRD = −0.02
MARD = 0.2
r = 0.92
MRD = 0.04
MARD = 0.3
r > 0.99
MRD = −0.03
MARD = 0.04
log10 (Salmon counts + 1)
log10 (Simulated counts + 1)
count
50
10
1

g

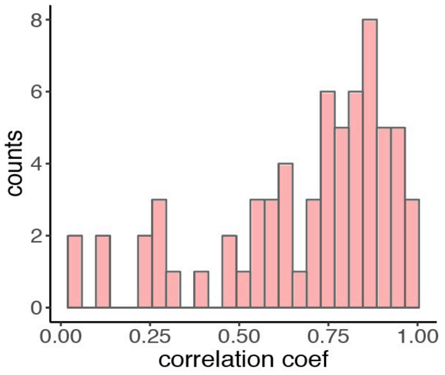
counts
correlation coef
0.00
0.25
0.50
0.75
1.00
0
2
4
6
8

h

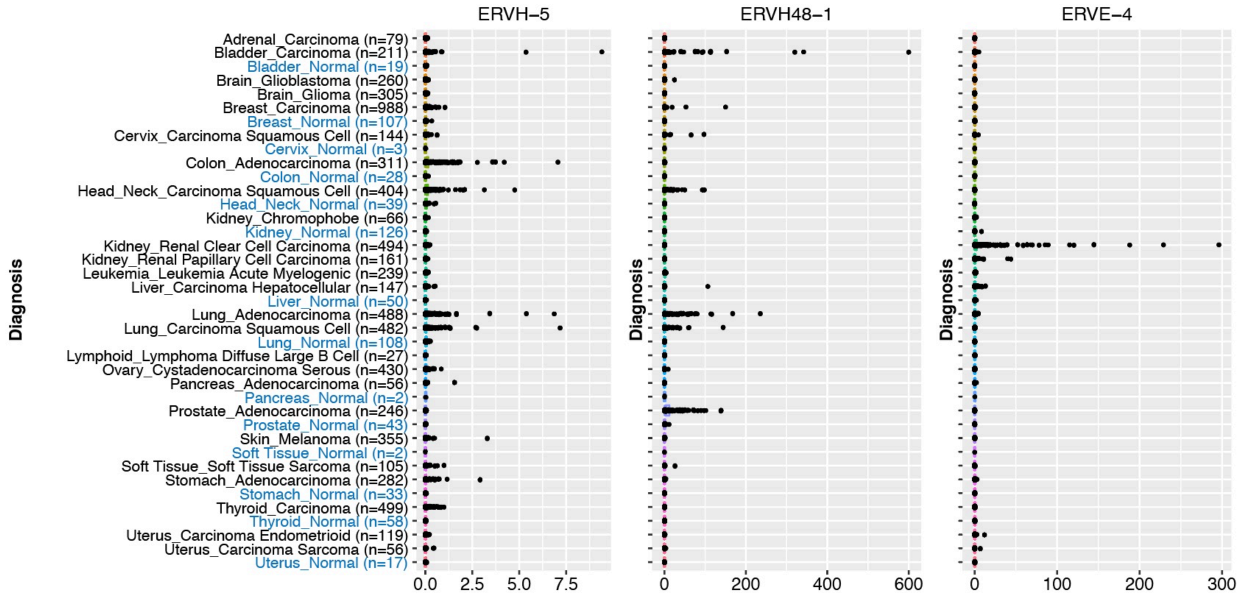
ERVH−5
ERVH48−1
ERVE−4
Diagnosis
Adrenal_Carcinoma (n=79)
Bladder_Carcinoma (n=211)
Bladder_Normal (n=19)
Brain_Glioblastoma (n=260)
Brain_Glioma (n=305)
Breast_Carcinoma (n=988)
Breast_Normal (n=107)
Cervix_Carcinoma Squamous Cell (n=144)
Cervix_Normal (n=3)
Colon_Adenocarcinoma (n=311)
Colon_Normal (n=28)
Head_Neck_Carcinoma Squamous Cell (n=404)
Head_Neck_Normal (n=39)
Kidney_Chromophobe (n=66)
Kidney_Normal (n=126)
Kidney_Renal Clear Cell Carcinoma (n=494)
Kidney_Renal Papillary Cell Carcinoma (n=161)
Leukemia_Leukemia Acute Myelogenic (n=239)
Liver_Carcinoma Hepatocellular (n=147)
Liver_Normal (n=50)
Lung_Adenocarcinoma (n=488)
Lung_Carcinoma Squamous Cell (n=482)
Lung_Normal (n=108)
Lymphoid_Lymphoma Diffuse Large B Cell (n=27)
Ovary_Cystadenocarcinoma Serous (n=430)
Pancreas_Adenocarcinoma (n=56)
Pancreas_Normal (n=2)
Prostate_Adenocarcinoma (n=246)
Prostate_Normal (n=43)
Skin_Melanoma (n=355)
Soft Tissue_Normal (n=2)
Soft Tissue_Soft Tissue Sarcoma (n=105)
Stomach_Adenocarcinoma (n=282)
Stomach_Normal (n=33)
Thyroid_Carcinoma (n=499)
Thyroid_Normal (n=58)
Uterus_Carcinoma Endometrioid (n=119)
Uterus_Carcinoma Sarcoma (n=56)
Uterus_Normal (n=17)
0.0
2.5
5.0
7.5
0
200
400
600
0
100
200
300

i

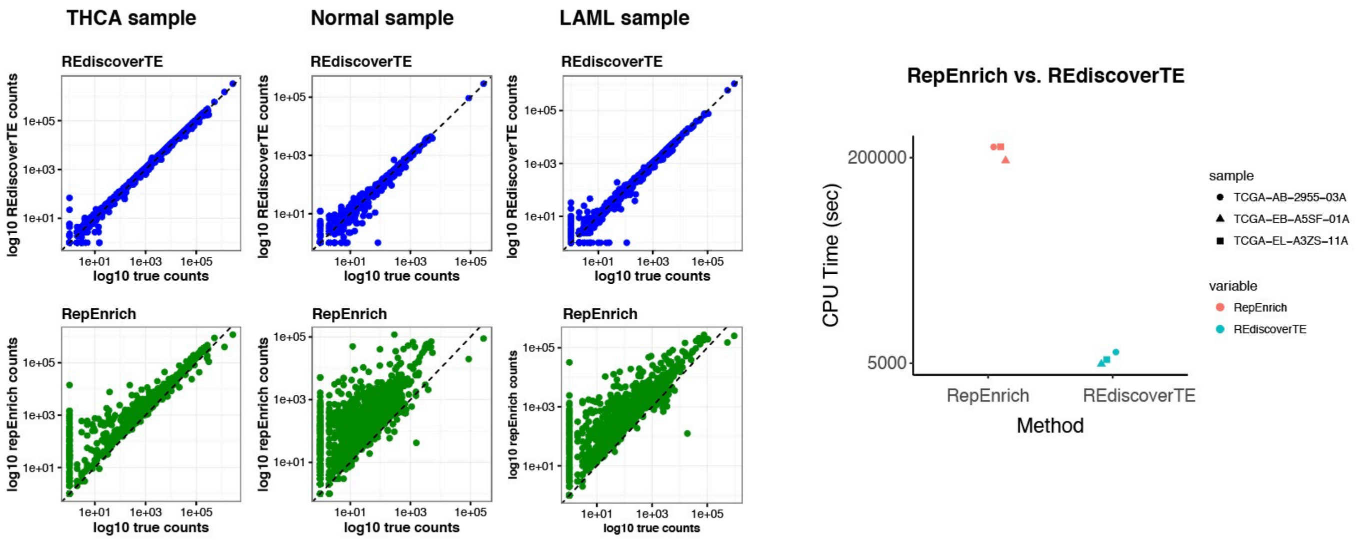
THCA sample
Normal sample
LAML sample
REdiscoverTE
RepEnrich
log10 REdiscoverTE counts
log10 repEnrich counts
log10 true counts
RepEnrich vs. REdiscoverTE
CPU Time (sec)
200000
5000
Method
RepEnrich
REdiscoverTE
sample
TCGA–AB–2955–03A
TCGA–EB–A5SF–01A
TCGA–EL–A3ZS–11A
variable
RepEnrich
REdiscoverTE

j

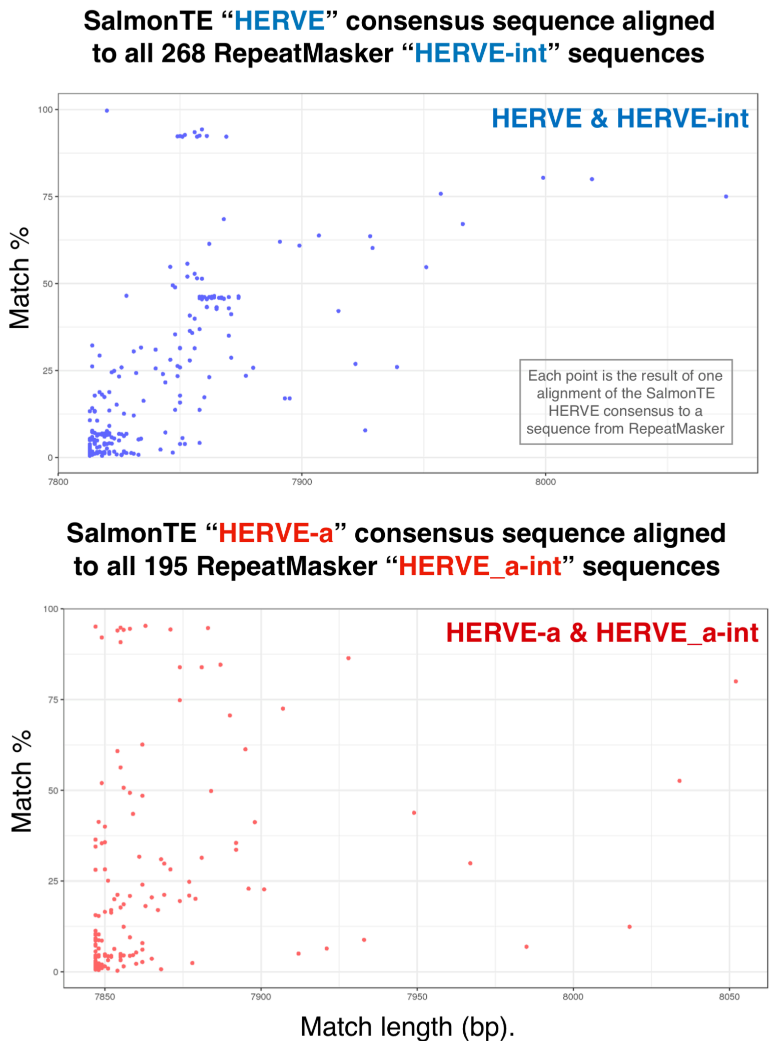
SalmonTE "HERVE" consensus sequence aligned to all 268 RepeatMasker "HERVE-int" sequences
HERVE & HERVE-int
Match %
Each point is the result of one alignment of the SalmonTE HERVE consensus to a sequence from RepeatMasker
SalmonTE "HERVE-a" consensus sequence aligned to all 195 RepeatMasker "HERVE_a-int" sequences
HERVE-a & HERVE_a-int
Match length (bp).

k

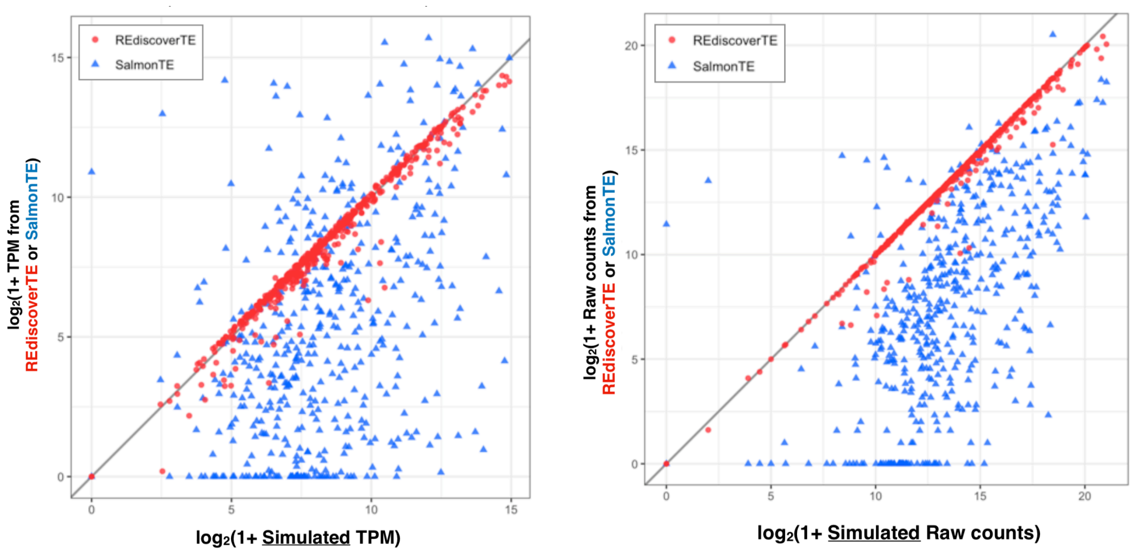

Supplementary Figure 1. Overview of the human repetitive genome and benchmarking *REdiscoverTE*

a. Repeatmasker hierarchical classification (class, family and subfamilies) of human repetitive DNA. Repetitive elements (RE) can be categorized into transposable elements (TE, left) and non-transposable elements (right). Numbers to the right are total counts of subfamilies within each class.

b. Abundance of repetitive elements in the human genome and their physical locations in relations to host genes defined in Gencode (v26 basic). Upper pie-chart: the content of RE in human genome according to the Human Repeatmasker (hg38). Lower pie-chart: the relative footprint of genomic features (CDs, UTRs, introns, noncoding genes) defined in the Gencode transcriptome. Regions outside Gencode features are considered intergenic. Bar-plot: enrichment of RE DNA in different genomic context in terms of Gencode genomic features. Numerical score of 1 corresponds to no enrichment, <1 corresponds to depletion, >1 corresponds to enrichment.

c. *REdiscoverTE* benchmarking workflow: 1) Generate transcriptome for RSEM simulation; 2) RSEM learns of expression pattern from real RNA-seq data; 3) RSEM simulates new RNA-seq fastq based on learned and adjusted statistics; 4) Salmon quantification of RSEM simulated fastq; 5) Evaluate Salmon's performance. This workflow was carried out for two TCGA samples: one LAML sample and one THCA sample. TPM: transcript per kilobase million. Venn diagram on physical locations of RE DNA in relations to genes for all RE subfamilies except those that belong to the class of simple repeats. 1,135 of these RE subfamilies have elements located in all 3 genomic regions (exon, intron, intergenic).

d. Post-hoc profiling of RE-to-transcript abundance in simulated data. Left: distribution of exonic RE to transcript TPM fold change for transcripts containing REs. Right: distribution of intron retention rate. Red: LAML sample. Blue: THCA sample.

e. Accuracy of *REdiscoverTE* RE quantification: TPM vs. counts. Top: simulation based on a TCGA LAML sample. Bottom: simulation based on a TCGA THCA sample. Left two panels: simulated TPM vs. estimated TPM. Right two panels: simulated read counts vs. estimated read counts. Index #1: reference transcriptome without inclusion of introns. Index #2: reference transcriptome that includes all introns containing REs. Performance accuracy is measured in terms of Spearman correlation coefficient (r), mean relative difference (MRD), mean absolute relative difference (MARD).

f. Accuracy of *REdiscoverTE* RE quantification with counts aggregated to the subfamily level. Top: simulation based on a TCGA LAML sample. Bottom: simulation based on a TCGA THCA sample.

g. Distribution of coefficients from Pearson correlation between *REdiscoverTE* and Rooney et al. Cell 2015 quantifications of 66 HERVs.

h. *REdiscoverTE* quantification of expression of 3 HERVs in TCGA RNA-seq data (compare to Rooney et al. Cell 2015 Fig4A)

i. Left 6 panels: comparison of TE quantification by *REdiscoverTE* to *RepEnrich*. Each point is one subfamily. Right panel: compute time in seconds used by *REdiscoverTE* vs. *RepEnrich* when quantifying the same fastq files using the same computer and memory resources.

j. Example illustrating that a single 'consensus' sequence can be a poor representative of all possible sequences in a subfamily that occur in a genome. Top: results of Needleman-Wunsch global alignment (via the software needle) of the HERVE sequence in the SalmonTE reference to all 268 *HERVE-int* sequences in RepeatMasker (hg38) as a function of their sequence lengths. Bottom: global alignment of the HERVE-a sequence in the SalmonTE reference to all 195 *HERVE_a-int* sequences in RepeatMasker as a function of their sequence lengths.

k. Comparison of *REdiscoverTE* and *SalmonTE* results to simulated ground truth on 474 TE subfamilies. Units of results are in log2 TPM (left) and counts (right) respectively. readouts

Supplementary Figure 2 (Related to Figure 2)

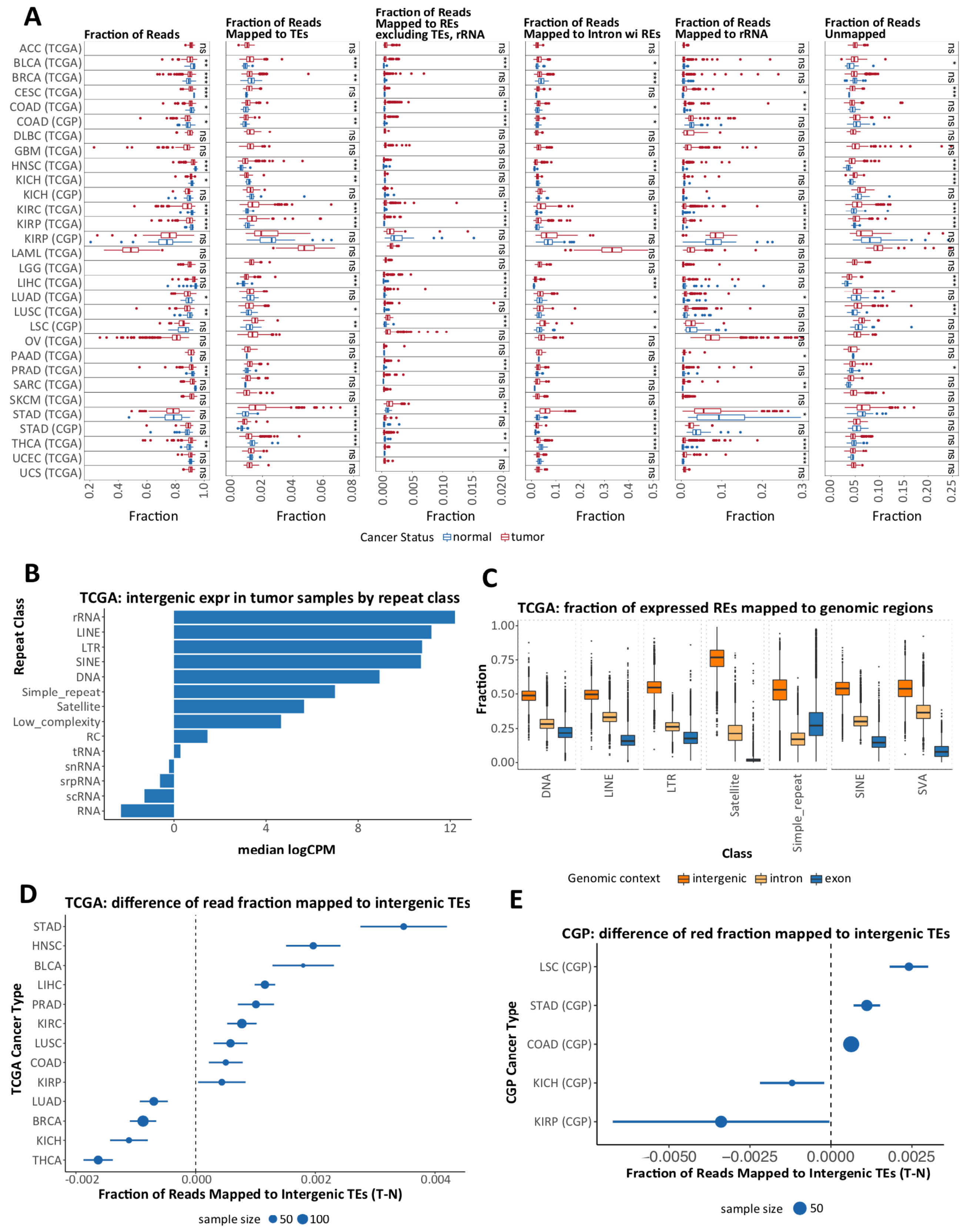

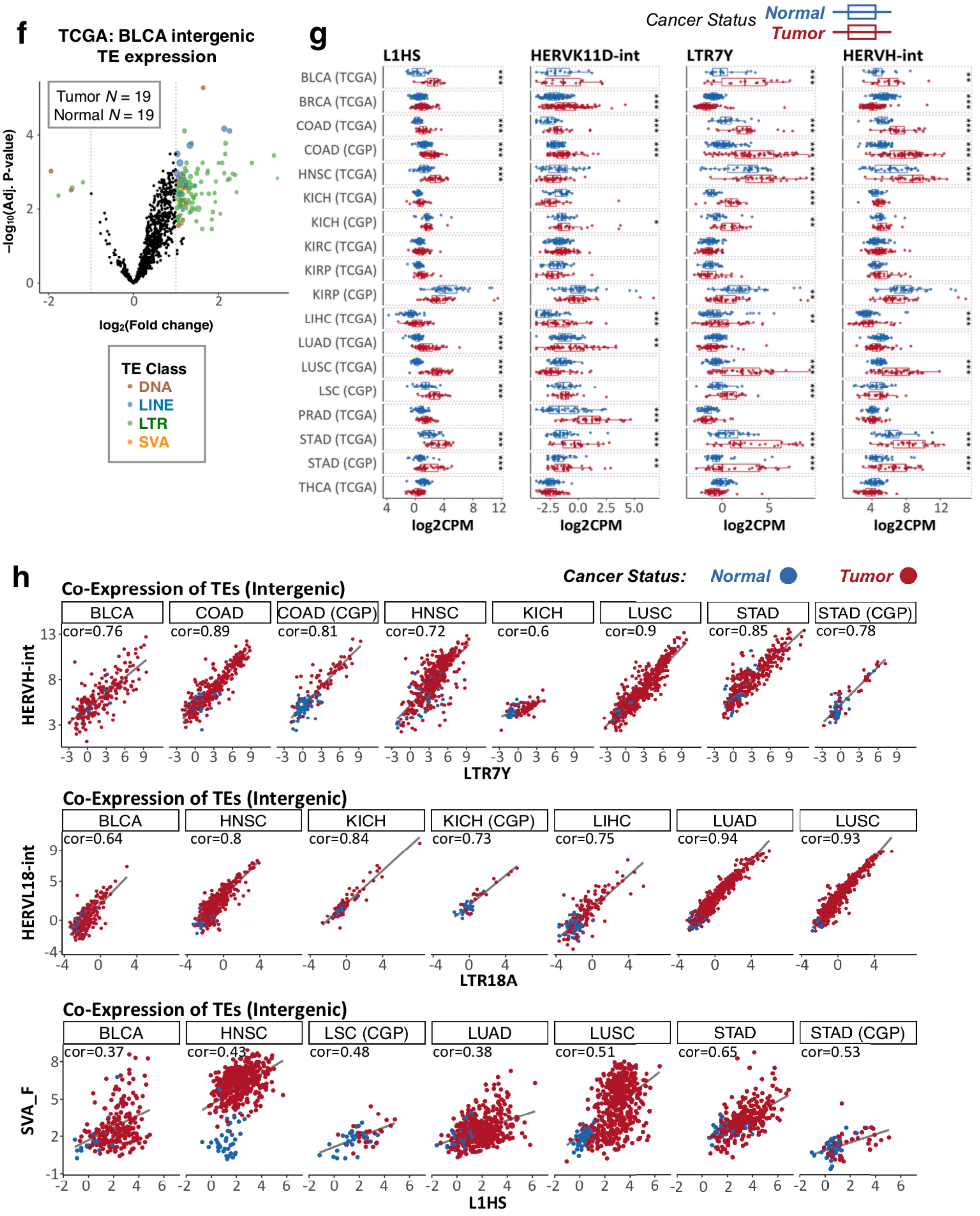

Supplementary Figure 2. Characteristics of TE expression in cancer

a. Fractions of TCGA and CGP RNAseq (both are poly-A preps) reads mapping to all features in the *REdiscoverTE* transcriptome (left to right): Gencode v26 basic transcripts,

Repeatmasker TEs (across cancer types: median 1.1%, mean 1.3%), Repeatmasker REs (excluding TEs, rRNAs), Gencode RE-containing introns and rRNAs. The last column is the fraction of reads that remained unmapped. Boxplot centerlines denote median and bounds denote first and third quartiles.

b. RE intergenic expression in TCGA tumor samples from distinct repeat classes.

c. Genomic context of RE expression for top 7 non-rRNA repeat classes in Supplementary Figure 2B. All TCGA samples are used for this calculation. For each repeat class, the denominator is total number of reads mapped to that class.

d. Difference in fractions of RNAseq reads mapped to Intergenic TEs in tumor samples compared to matched normal samples across 13 TCGA cancer types (each with at least 10 normal sample). Error bars are standard errors of the mean.

e. Same as Supplementary Figure 2D, except for the 5 CGP cancer types. Error bars are standard errors of the mean.

f. Example volcano plots of intergenic TE differential expression aggregated to the subfamily level performed on TCGA BLCA, 19 tumor and matched normal samples.

g. Patterns of differential expression for 4 TE subfamilies consistently over-expressed across cancer types in both TCGA and CGP. Only tumor and normal sample pairs are included here. Red: tumor samples. Blue: matched normal samples. Asterisks indicate level of significance in differential expression analysis between tumor and matched normal: * abs(log2 fold change) > 1 & FDR<0.05, ** abs(log2 fold change) > 1 & FDR<0.01, *** abs(log2 fold change) > 1 & FDR<0.001

h. Co-expression of three pairs of over-expressed TEs. Units in log2 CPM. Red: all tumor samples. Blue: available matched normal samples.

Supplementary Figure 3 (Related to Figure 3)

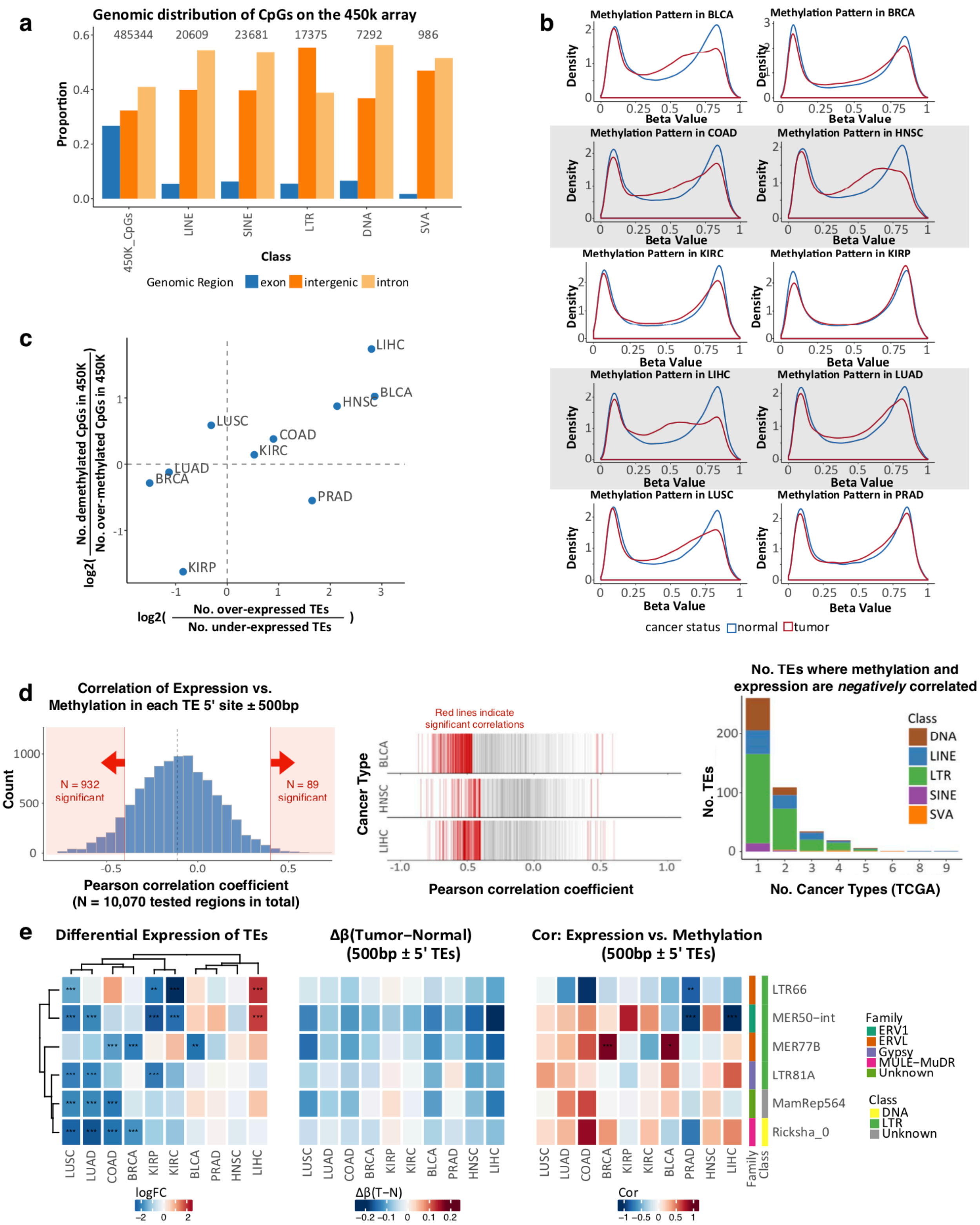

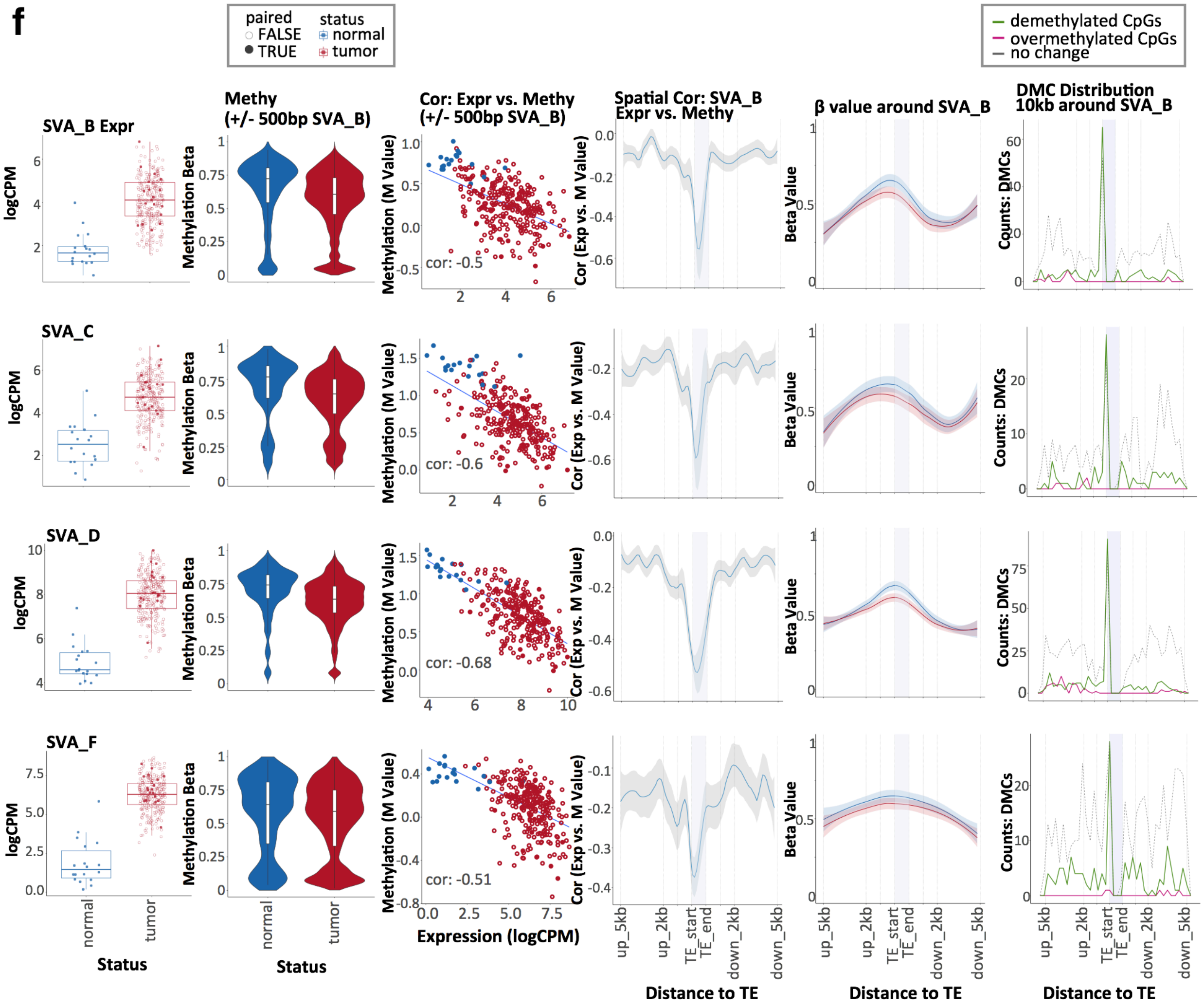

Supplementary Figure 3. Association between TE expression and loss of DNA methylation in cancer

a. Illumina 450K array coverage of CpG sites in relations to TE (by class) as well as to genes. Each set of 3 bars represent the fraction of all CpGs, either in its entirety (450K_CpGs) or overlapping with a particular TE class (e.g. LINE) grouped by their physical location in relations to Gencode host genes (exon, intron and intergenic regions).

b. Global distribution of beta values of all 450k CpGs for 10 TCGA cancer types with methylation data. Only matched tumor-normal samples are used. Red: tumor samples. Blue: matched normal samples.

c. The extent of TE mRNA over-expression is strongly correlated with the extent of global CpG demethylation across cancer types.

d. Distribution coefficients of Pearson correlation between intergenic TE expression (N=1007 subfamilies) with average CpG methylation (M-value averaged over 500bp+/- 5' bp of the corresponding TEs) using matched tumor-normal samples across 10 cancer types. Significance threshold: cor < abs(0.4) and BH FDR<0.05. Left: pooled correlation coefficients for 10 TCGA cancer types. Median cor=-0.11. There are 932 significant inverse correlations and 89 positive correlations across the 10 cancer types. Middle: correlation coefficients for 3 most de-methylated cancer types: BLCA, HNSC, LIHC. Red lines indicate significant correlations. Right: Across 10 cancer types there were 431 unique TE subfamilies with significant inverse correlation between expression and methylation. Some TEs show inverse correlation in multiple cancer types. Histogram shows distribution on the recurrence of these inverse correlation.

e. Examples of TE subfamilies with reduced-expression in tumor compared to matched normal and their CpG methylation status. Selection criteria: TE subfamilies showed significantly reduced expression in >=3 cancer types. Left: log2 FC values of tumor vs. normal differential expression of TEs (row) across cancer types (column). Middle: tumor - normal delta beta value at CpG 500bp+/- 5'bp of TE locations. Right: Correlation between intergenic TE expression and methylation M value at CpG 500bp +/- 5'bp of TE locations.

f. Examples from HNSC: expressions of 4 SVA subfamilies are associated with DNA methylation status. Blue: normal sample. Red: tumor samples. Filled circle: tumor samples with matched normal. Open circle tumor samples without matched normal. Grey shading: 95% confidence interval. Column 1: normal and tumor SVA intergenic expression. Column 2: normal and tumor CpG beta values in 500bp+/- around 5'bp of intergenic SVA. Column 3: correlation between SVA intergenic expression and methylation M value (500bp+/- 5'bp intergenic SVA). Column 4: spatial correlation between intergenic SVA expression and CpG methylation M value around 5kb +/- SVAs. SVA gene body is shaded in blue. Column 5: smoothed beta value in tumor and matched normal pairs in 5kb +/- region around SVA. Column 6: spatial distribution of differentially methylated cytosines (DMCs): demethylated CpG sites (green), over-methylated CpG sites(magenta) and CpGs with no change (grey, dashed) around 5kb +/- SVAs. Boxplot centerlines denote median and bounds denote first and third quartiles.

Supplementary Figure 4 (Related to Figure 4)

a

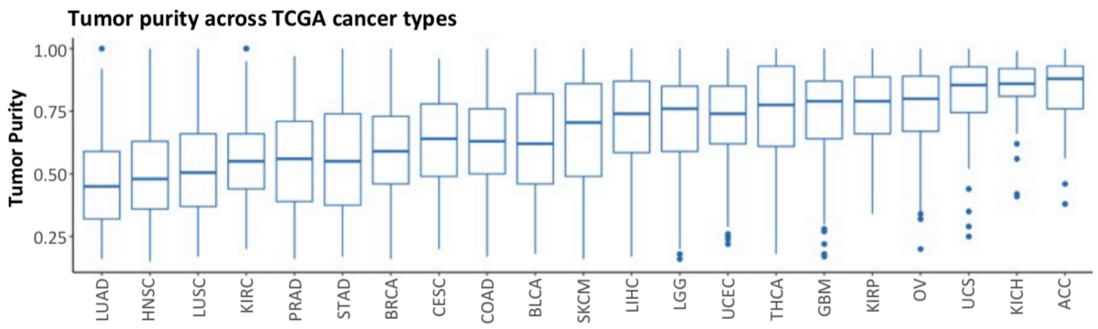

b

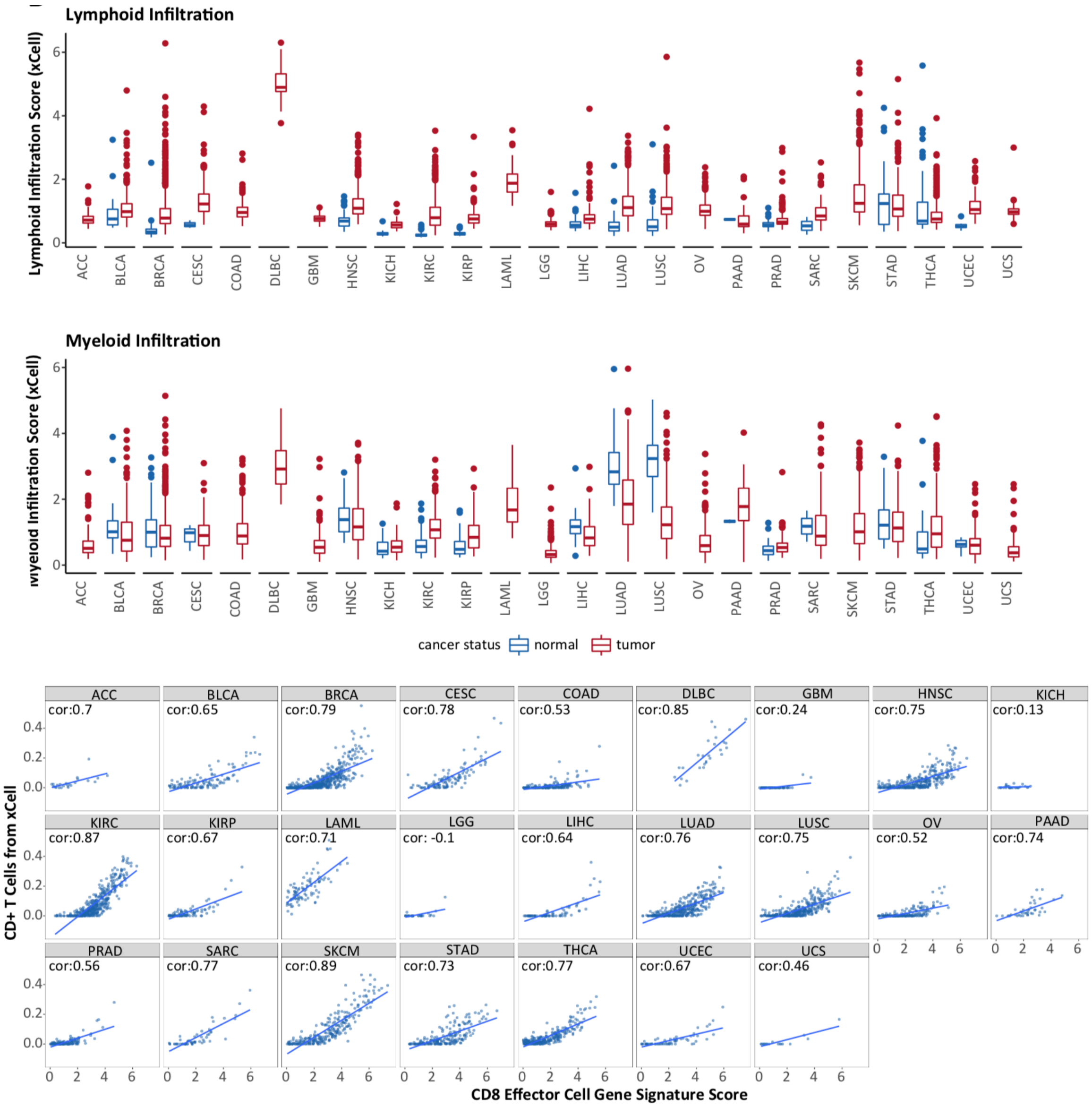

c

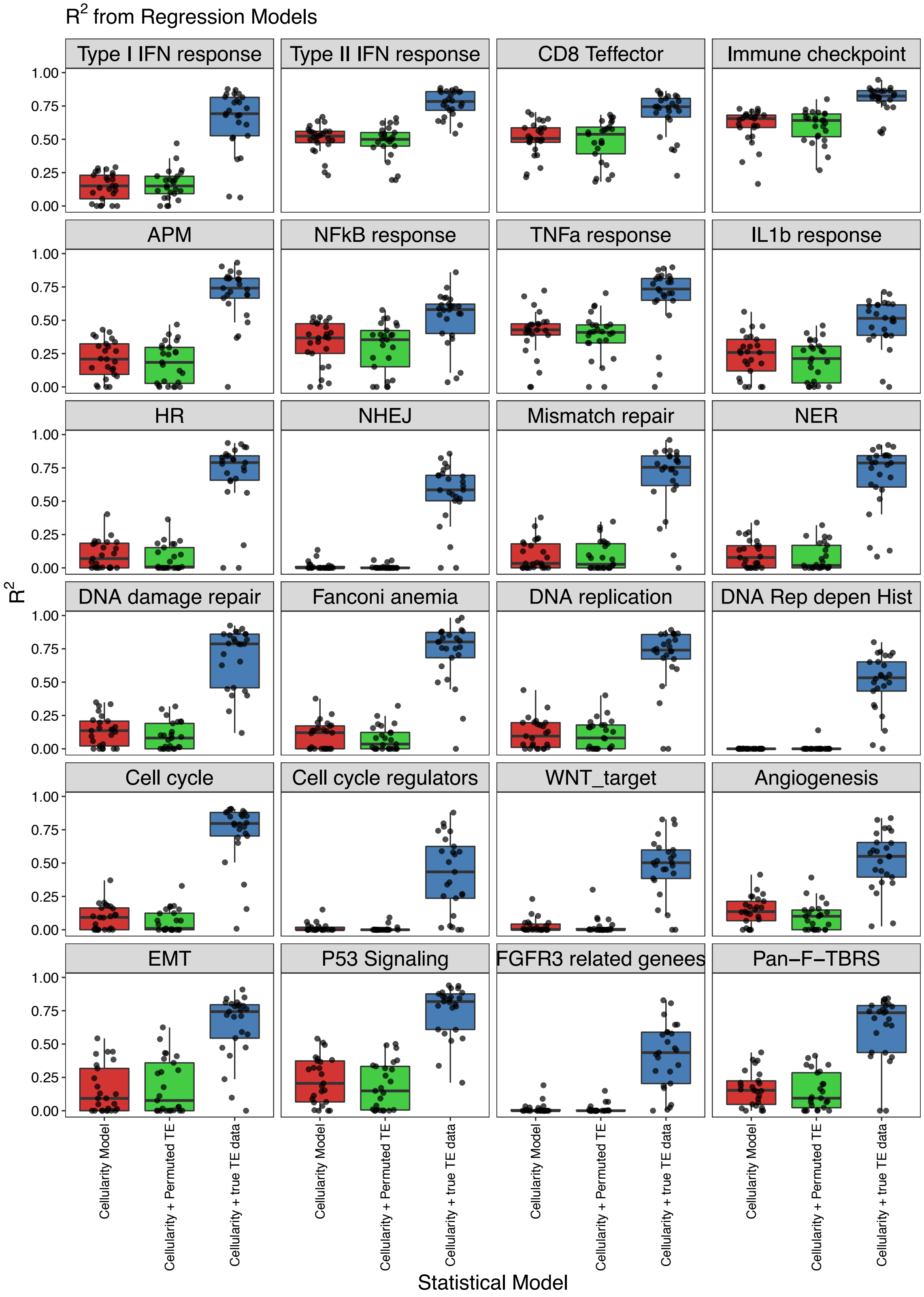

R² from Regression Models
Type I IFN response
Type II IFN response
CD8 Teffector
Immune checkpoint
APM
NFkB response
TNFa response
IL1b response
HR
NHEJ
Mismatch repair
NER
DNA damage repair
Fanconi anemia
DNA replication
DNA Rep depen Hist
Cell cycle
Cell cycle regulators
WNT_target
Angiogenesis
EMT
P53 Signaling
FGFR3 related genees
Pan−F−TBRS
R²
1.00
0.75
0.50
0.25
0.00
Cellularity Model
Cellularity + Permuted TE
Cellularity + true TE data
Statistical Model

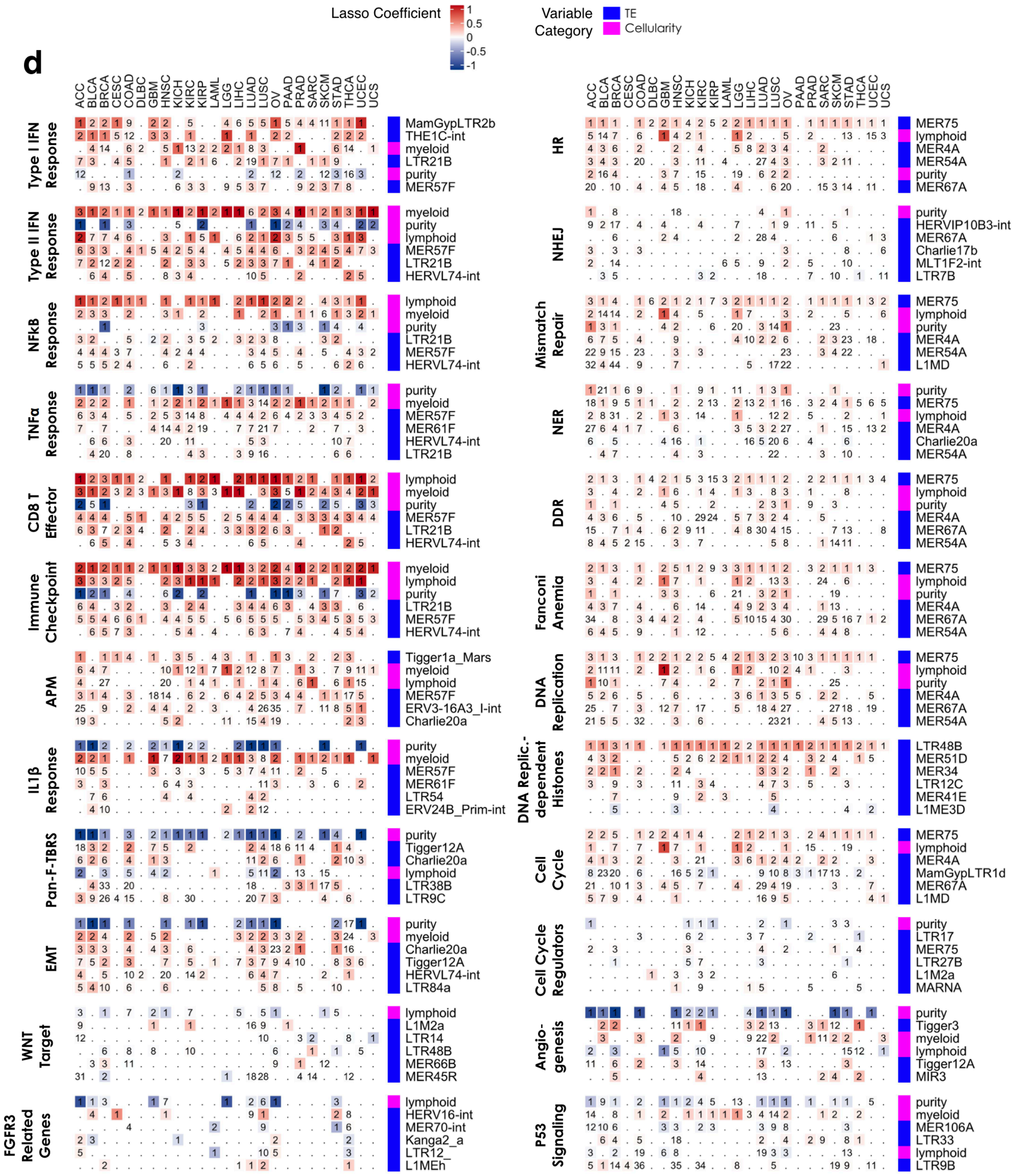

Lasso Coefficient
Variable Category
TE
Cellularity
d
Type I IFN Response
Type II IFN Response
NFkB Response
TNFα Response
CD8 T Effector
Immune Checkpoint
APM
IL1β Response
Pan-F-TBRS
EMT
WNT Target
FGFR3 Related Genes
HR
NHEJ
Mismatch Repair
NER
DDR
Fanconi Anemia
DNA Replication
DNA Replic.-dependent Histones
Cell Cycle
Cell Cycle Regulators
Angio-genesis
P53 Signaling

e

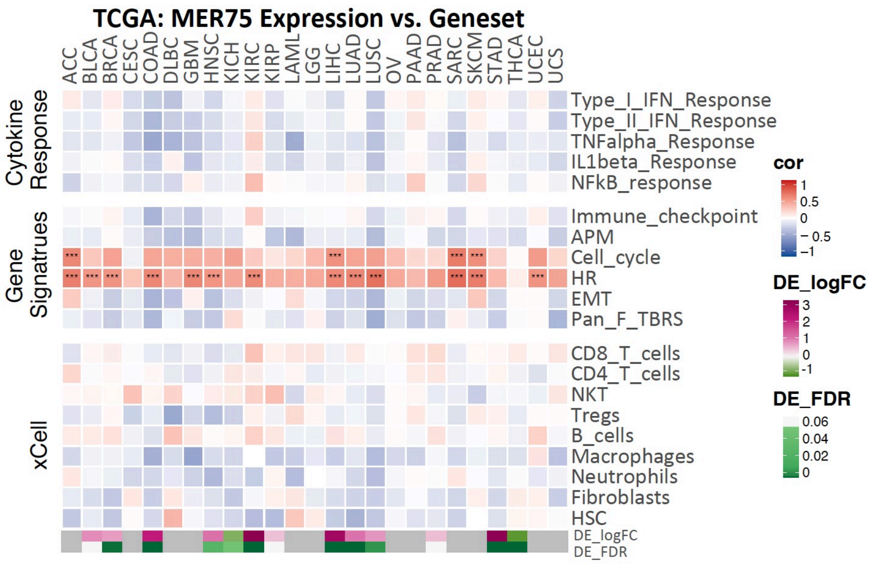
TCGA: MER75 Expression vs. Geneset
ACC
BLCA
BRCA
CESC
COAD
DLBC
GBM
HNSC
KICH
KIRC
KIRP
LAML
LGG
LIHC
LUAD
LUSC
OV
PAAD
PRAD
SARC
SKCM
STAD
THCA
UCEC
UCS
Cytokine Response
Type_I_IFN_Response
Type_II_IFN_Response
TNFalpha_Response
IL1beta_Response
NFkB_response
Gene Signatrues
Immune_checkpoint
APM
Cell_cycle
HR
EMT
Pan_F_TBRS
xCell
CD8_T_cells
CD4_T_cells
NKT
Tregs
B_cells
Macrophages
Neutrophils
Fibroblasts
HSC
DE_logFC
DE_FDR
cor
1
0.5
0
− 0.5
−1
DE_logFC
3
2
1
0
-1
DE_FDR
0.06
0.04
0.02
0

f

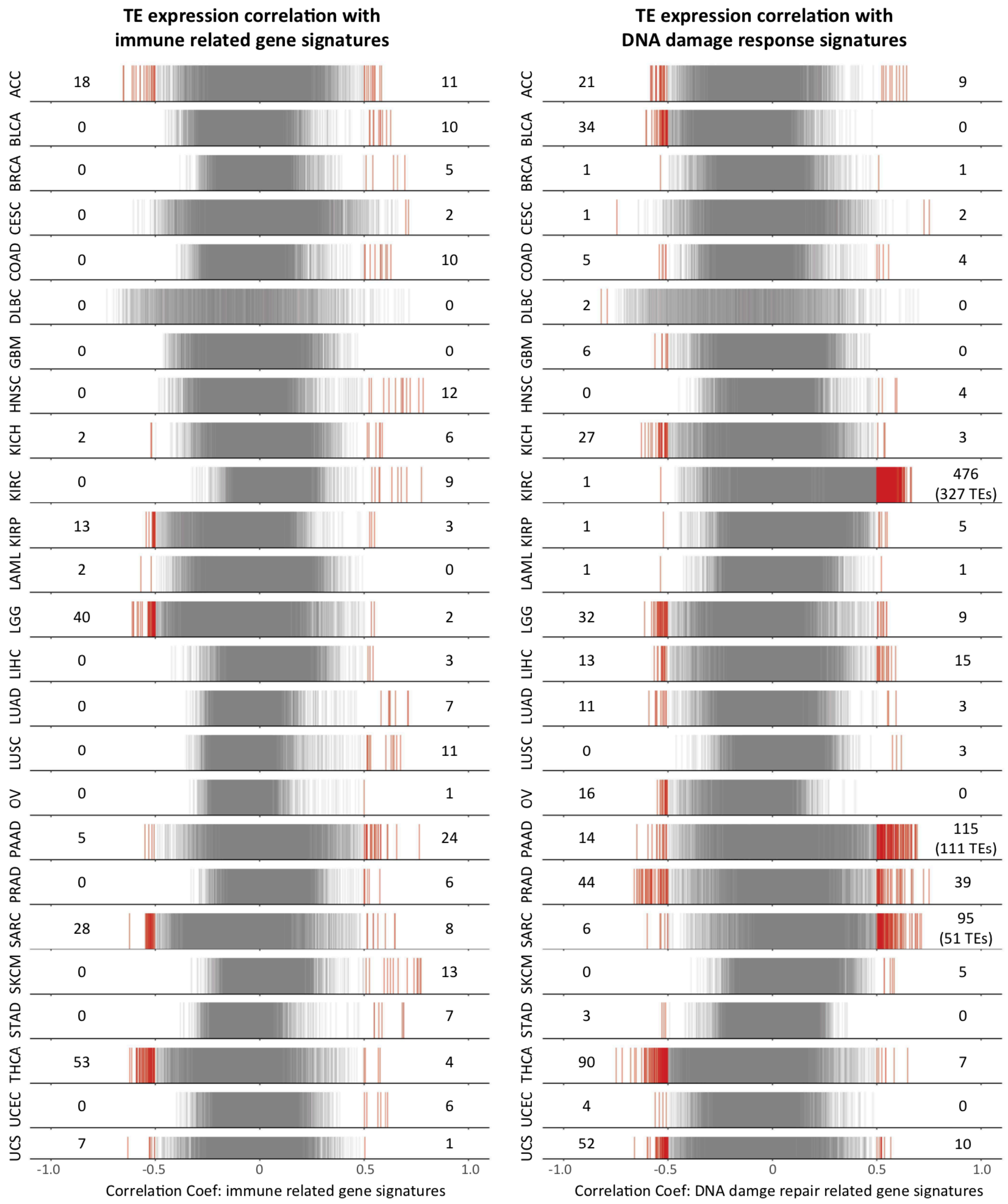
TE expression correlation with immune related gene signatures
TE expression correlation with DNA damage response signatures
ACC 18 11
BLCA 0 10
BRCA 0 5
CESC 0 2
COAD 0 10
DLBC 0 0
GBM 0 0
HNSC 0 12
KICH 2 6
KIRC 0 9
KIRP 13 3
LAML 2 0
LGG 40 2
LIHC 0 3
LUAD 0 7
LUSC 0 11
OV 0 1
PAAD 5 24
PRAD 0 6
SARC 28 8
SKCM 0 13
STAD 0 7
THCA 53 4
UCEC 0 6
UCS 7 1
ACC 21 9
BLCA 34 0
BRCA 1 1
CESC 1 2
COAD 5 4
DLBC 2 0
GBM 6 0
HNSC 0 4
KICH 27 3
KIRC 1 476 (327 TEs)
KIRP 1 5
LAML 1 1
LGG 32 9
LIHC 13 15
LUAD 11 3
LUSC 0 3
OV 16 0
PAAD 14 115 (111 TEs)
PRAD 44 39
SARC 6 95 (51 TEs)
SKCM 0 5
STAD 3 0
THCA 90 7
UCEC 4 0
UCS 52 10
-1.0
-0.5
0
0.5
1.0
Correlation Coef: immune related gene signatures
Correlation Coef: DNA damge repair related gene signatures

g

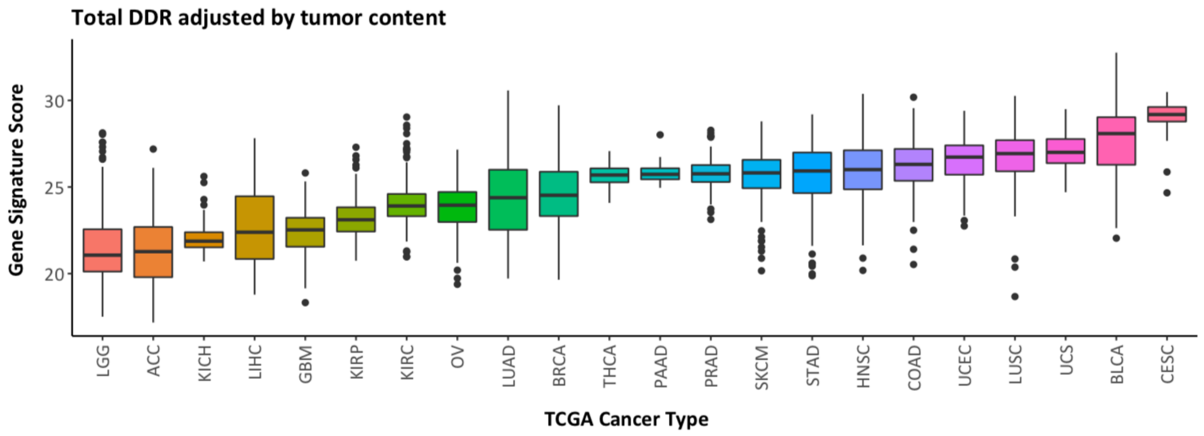

Supplementary Figure 4. Characteristics of tumor gene expression profile in relations to TE

a. Distribution of tumor purity score for TCGA samples by cancer type. Boxplot centerlines denote median and bounds denote first and third quartiles.

b. Top panel and middle panels: total lymphoid and myeloid abundance in TCGA tumors, respectively, estimated based on xCell. Total lymphoid score is the sum of xCell scores of CD8+ T-cells, NK cells, CD4+ naive T-cells, B-cells, CD4+ T-cells, CD8+ Tem, Tregs, plasma cells, CD4+ Tcm, CD4+ Tem, memory B-cells, CD8+ Tcm, naive B-cells, CD4+ memory T-cells, pro B-cells, class-switched memory B-cells, Th2 cells, Th1 cells, CD8+ naive T-cells, NKT and Tgd cells. Total myeloid score is the sum of xCell scores of monocytes, macrophages, DC, neutrophils, eosinophils, macrophages M1, macrophages M2, aDC, basophils, cDC, pDC, iDC, mast cells. Bottom panel: correlation between xCell CD8+ T cell score and CD8+ Effector T cell geneset score estimated from multiGSEA. Each panel is one cancer type; each point is one sample. Samples with xCell score of 0 were omitted. Boxplot centerlines denote median and bounds denote first and third quartiles.

c. R^2 values from 3 regression models on 24 gene signatures in 25 cancer types. Each panel is one gene signature, each point is one cancer type. Red: R^2 from cellularity linear model which includes tumor content, total lymphoid and myeloid scores as predictors. Blue: R^2 of Lasso model which includes 3 aforementioned cellularity parameters and expression level of all 1,052 TEs as predictors. Green: R^2 from linear model taking top 6 TEs predicted by Lasso model and 3 cellularity parameters as predictors. Boxplot centerlines denote median and bounds denote first and third quartiles.

d. Graphical overview of top hits from Lasso models of gene signature scores across 25 TCGA cancer types. Only the top 6 variables predicting the gene signatures are included in the heatmaps. Top variables were selected based on rank order of the number of cancer types in which a given variable (e.g. a *L1HS*) had non-zero coefficients. Heatmap colors denote the value of the coefficient from Lasso model -- red and blue correspond to

positive and negative coefficient values, respectively. Dots in the heatmap denote zero coefficients assigned by Lasso. Numbers in the heatmap indicate the rank of the absolute value of non-zero coefficients from the Lasso model for a given cancer type. Side bar colors indicate whether a variable is a TE subfamily (blue) or one of three cellularities (magenta).

e. Heatmap showing association between MER75 expression and gene signatures as well as immune infiltrates estimated by xCell across 25 cancer types. MER75 expression is strongly associated with and DNA damage as well as cell cycle. Heatmap colors denote Spearman correlation coefficient. Differential expression status is denoted at the bottom. *** FDR<0.001, BH corrected.

f. Distribution of coefficients from Spearman correlations between the expression of 1052 TE subfamilies and gene signature scores across 25 TCGA cancer types. Left: pooled correlation coefficients from correlations with 8 immune related gene signatures (Type I IFN Response, Type II IFN Response, NFkB, TNFalpha, CD8 T Effector, Immune checkpoint, Antigen Processing Machinery, IL1b Response)
Right: pooled correlation coefficients from correlations with 6 DNA damage related gene signatures (NHEJ, Homologous Recombination, Mismatch repair, Nucleotide excision repair, DNA damage repair, Fanconi anemia). Correlation was calculated using only tumor samples and controlled for tumor content. Red: significant and strong correlations. Significance threshold: abs(cor)>0.5 & FDR<0.05, BH corrected. Numbers indicate the number of significant correlations except those in parenthesis indicate number of unique TE subfamilies with significant correlations.

g. Distribution of tumor DNA damage response (DDR) scores, adjusted for tumor purity scores. DDR scores are computed as sum of 6 DNA damage related gene signature scores: homologous recombination, NHEJ, DNA damage repair, Fanconi anemia, nucleotide excision repair and mismatch repair. Adjusted total DDR is the intercept plus residual of linear regression of total DDR score on tumor purity score. Boxplot centerlines denote median and bounds denote first and third quartiles.

Supplementary Figure 5 (Related to Figure 5)

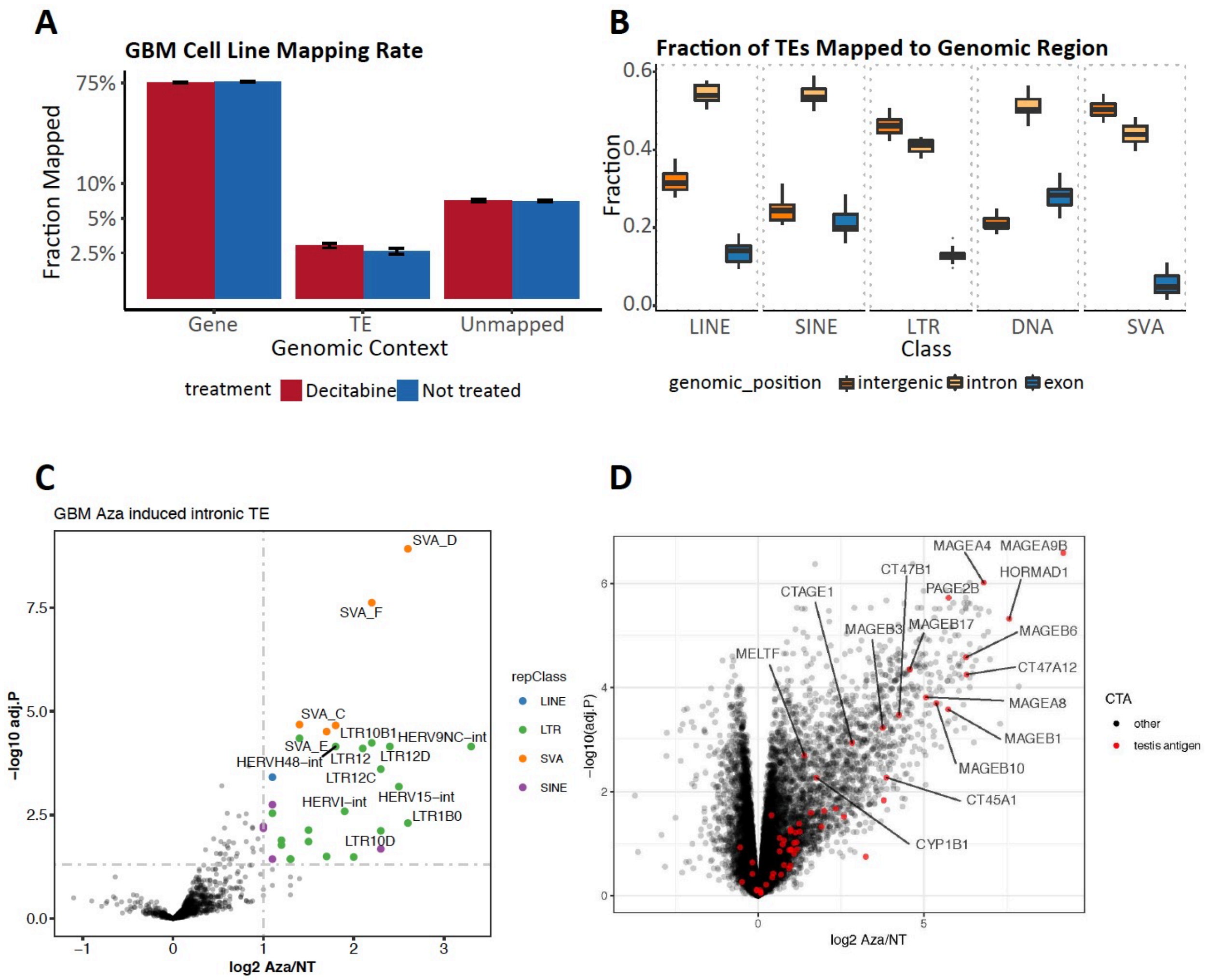

Supplementary Figure 5. Decitabine treatment of GBM cell lines induces TE expression

a. Fractions of RNA-seq output (rRNA depletion prep) corresponding to reads mapped to Gencode genes, Repeatmasker TEs and unmapped reads. Error bars denote standard error over 12 samples.

b. Fraction of RNA-seq reads mapped to intergenic, intronic and exonic TE elements for each of 5 TE classes. Boxplot centerlines denote median and bounds denote first and third quartiles.

c. Volcano plot showing differential intronic expression of TE subfamilies, decitabine-treated (Aza) vs. non-treated (NT). TE subfamilies are colored by class at the significance threshold of log2FC > 1 and adjusted p-value < 0.05 and labeled if log2FC>1.5 and adjust p-value < 0.01.

d. Decitabine treatment results in many cancer testis antigens. Aza vs. NT volcano plots showing differential expression of Gencode genes. Red: select cancer testis antigens.

Supplementary Tables

Supplementary Table 1. REdiscoverTE human transcriptome (GRCh38 assembly, Gencode v26 basic)

Annotated Genomic Features (GRCh38)	Number of features
**Human transcripts (Gencode v26 Basic)**	98,029
**Introns containing REs**	185,403
**RepeatMasker RE sequences***	5,099,056
**RE located in exons****	86,262
**RE located in introns**	2,575,623
**RE located in intergenic regions**	2,437,171

* unique sequences, poly-A sequences removed
** for REs overlapping multiple contexts, their locations are assigned with the following priority: exon > intron > intergenic. For example, a RE overlapping both exon and intron is counted as exonic RE

Supplementary Table 2. TCGA and CGP RNAseq sample

Counts of TCGA tumor and matched normal RNAseq samples. Total N = 7,345

TCGA CANCER TYPES	CANCER NAME	MATCHED TUMOR	MATCHED NORMAL	TUMOR SAMPLES W/O MATCHED NORMAL
**ACC**	Adrenocortical carcinoma	0	0	79
**BLCA**	Bladder Urothelial Carcinoma	19	19	192
**BRCA**	Breast invasive carcinoma	109	107	879
**CESC**	Cervical squamous cell carcinoma and endocervical adenocarcinoma	3	3	141
**COAD**	Colon adenocarcinoma	26	26	285
**DLBC**	Lymphoid Neoplasm Diffuse Large B-cell Lymphoma	0	0	27
**GBM**	Glioblastoma multiforme	0	0	170
**HNSC**	Head and Neck squamous cell carcinoma	36	36	368
**KICH**	Kidney Chromophobe	25	25	41

**KIRC**	Kidney renal clear cell carcinoma	71	71	423
**KIRP**	Kidney renal papillary cell carcinoma	30	30	131
**LAML**	Acute Myeloid Leukemia	0	0	123
**LGG**	Brain Lower Grade Glioma	0	0	305
**LIHC**	Liver hepatocellular carcinoma	50	50	97
**LUAD**	Lung adenocarcinoma	56	56	432
**LUSC**	Lung squamous cell carcinoma	50	50	432
**OV**	Ovarian serous cystadenocarcinoma	0	0	412
**PAAD**	Pancreatic adenocarcinoma	2	2	54
**PRAD**	Prostate adenocarcinoma	43	43	203
**SARC**	Sarcoma	2	2	103
**SKCM**	Skin Cutaneous Melanoma	0	0	355
**STAD**	Stomach adenocarcinoma	30	30	252
**THCA**	Thyroid carcinoma	58	58	441
**UCEC**	Uterine Corpus Endometrial Carcinoma	7	7	112
**UCS**	Uterine Carcinosarcoma	0	0	56
**TOTAL**		**617**	**615**	**6113**

Counts of CGP tumor and matched normal RNAseq samples. Total N = 405

**CGP CANCER TYPES**	**TUMOR**	**NORMAL**
**COLON ADENOCARCINOMA**	73	73
**LUNG SMALL CELL**	27	27
**KIDNEY CHROMOPHOBE**	25	25
**KIDNEY RENAL PAPILLARY CELL CARCINOMA**	41	40
**STOMACH ADENOCARCINOMA**	37	37
**TOTAL**	**203**	**202**

Supplementary Table 3. Summary of TCGA DNA methylation analysis

Cancer Type	Samples Normal/ Tumor	all DMC	All DMC % DEmethylatED	RE DMC	RE % DEmethylatED	RE CpG DEmethylation enrichment SCORE
**BLCA**	14/14	99,680	73.6%	18,497	92.6%	4.5
**BRCA**	74/75	93,971	43.0%	12,612	71.7%	3.4
**COAD**	6/6	67,557	59.5%	11,211	87.7%	4.8
**HNSC**	18/18	97,558	70.6%	18,936	91.9%	4.7
**KIRC**	22/22	70,613	53.7%	10,334	73.0%	2.3
**KIRP**	21/21	58,103	16.5%	6,282	32.3%	2.4
**LIHC**	30/30	130,350	85.1%	26,812	96.2%	4.4
**LUAD**	15/15	58,885	47.0%	7,676	78.1%	4.0
**LUSC**	7/7	78,275	64.4%	14,050	85.6%	3.3
**PRAD**	29/29	79,216	36.7%	11,224	64.6%	3.1

Only tumors with matched normal samples are included for analysis
DMC defined as average absolute beta value change from normal > 10% and FDR < 0.05
TE demethylation enrichment score defined in Methods

Supplementary Note 1

Here we provide additional information on *REdiscoverTE* performance benchmarking

Comparing *REdiscoverTE* to Rooney et al. Cell 2015

We performed a direct comparison of *REdiscoverTE* to a previously published study on a restricted set of 66 human endogenous retroviruses (HERVs) in TCGA by Rooney et al. (Rooney et al. 2015). This direct comparison was made by running Salmon using the same transcriptome as Rooney et al., which consisted of the human genic transcriptome and 124 sequences of HERVs. One major difference was that Rooney's approach was step-wise: quantification of genes first, then HERVs; our approach was simultaneous quantification. Comparing to cpm values published in Rooney et al. 2015 Supplementary Table 5b, we found our results were generally consistent (median r = 0.76, **Supplementary Figure 1g**), particularly for 3 previously identified tumor-specific HERVs: *ERVH-5*, *ERVH48-1* and *ERVE-4* (**Supplementary Figure 1h**).

However, *REdiscoverTE* by design has the advantage of capturing expression by far more REs in the transcriptome. When part of the transcriptome is missing from the reference, reads that would have mapped to the missing transcript models would either become unmapped or mis-assigned to transcripts in the reference. Our complete RE transcriptome enables more accurate assignment of RNAseq reads.

Comparing *REdiscoverTE* to *Repenrich*

We compared *REdiscoverTE* to *Repenrich* (Criscione et al. 2014), a two-step alignment-based TE expression quantification method that first aligns reads to host genes using Bowtie (Langmead et al. 2009), then quantifies TE expression. Benchmarking against simulated data, we showed that *REdiscoverTE* performed with higher accuracy and computational efficiency (**Supplementary Figures 1i**), while *RepEnrich* showed significant over-estimation of TE expression. Likely reasons for *RepEnrich*'s over-estimation include the addition of padding sequences in its TE reference and the assignment of reads that belong to genes and retained introns to overlapping TEs. *REdiscoverTE* overcomes these biases with the inclusion of intron sequences in its transcriptome and simultaneous host gene/RE quantification.

Comparing *REdiscoverTE* to *SalmonTE*

We compared *REdiscoverTE* to *SalmonTE* v0.4 (Jeong et al. 2018) which uses Salmon to quantify the expression of 687 human TEs. *SalmonTE*'s reference transcriptome is comprised of 687 TE sequences from Repbase. While these sequences may represent the consensus sequences for each of the 687 TE subfamilies, such a limited TE transcriptome cannot account for the extensive sequence diversity within each TE subfamily and cannot capture expression from TE loci that significantly deviate from the Repbase sequences. We illustrate this problem with two examples in **Supplementary Figure 1j**, where the sequence similarity (percent match) of a given instance of a HERV to its *SalmonTE*/Repbase sequence is often below 50%.

Another major distinction between *SalmonTE* and *REdiscoverTE* is that *SalmonTE* does not simultaneously quantify gene expression from the canonical human transcriptome. As we show in our manuscript, when mapping short read sequencing data, an incomplete representation of the

reference transcriptome can result in significant bias on TE expression readout due to sequence homology between certain TEs and canonical human genes. A direct comparison of the *REdiscoverTE* and *SalmonTE* reference transcriptomes is shown in the following table.

Method	Distinct RE sequences in the transcriptome	Aggregation to subfamilies	Human gene transcriptome	Human introns containing REs
REdiscoverTE	N ~ 5 Million	N = 1052	Gencode v26 basic (N ~ 98K)	~185K
SalmonTE	N = 687	No aggregation	None	None

As a direct comparison, we applied the two methods to a simulated RNAseq sample generated using RSEM based on the TCGA LAML tumor sample, TCGA-AB-2955-03A-01T-0734-13 (see Methods) where the 'ground truth' on expression levels for each TE subfamily is known. Among the 687 TE subfamilies used by *SalmonTE*, 474 are also present in the RepeatMasker database under identical names. Comparing to the simulated ground truth, *SalmonTE*'s readouts on these 474 TE subfamilies show significant under-estimation of expression, both in counts and TPM, while *REdiscoverTE*'s readouts were highly accurate for most of the TE subfamilies (**Supplementary Figure 1k**).
