# Supplementary material for: Transposable element expression in tumors is associated with immune infiltration and increased antigenicity
Source: Nat Commun. 2019 Nov 19;10:5228. doi: 10.1038/s41467-019-13035-2 (PMC6864081; doi:10.1038/s41467-019-13035-2)
Supplement: Supplementary file 2 — Description of Additional Supplementary Files [file 41467_2019_13035_MOESM2_ESM.pdf]

Description of Additional Supplementary Files

File Name: Supplementary Data 1

Description: TCGA and CGP TE differentially expression results

File Name: Supplementary Data 2

Description: TE subfamilies recurrently significantly over-expressed in multiple TCGA cancer

File Name: Supplementary Data 3

Description: TE subfamilies with expression inversely correlated with DNA methylation in multiple TCGA cancer types

File Name: Supplementary Data 4

Description: List of gene signatures related to tumors, immune response and DNA damage response

File Name: Supplementary Data 5

Description: Summary of Lasso regression and cellularity linear regression results

File Name: Supplementary Data 6

Description:Differentially expressed TE subfamilies in GBM cell lines upon decitabine treatment

File Name: Supplementary Data 7

Description: List of TE-derived peptides identified from mass spectroscopy in GBM cell lines

File Name: Supplementary Data 8

Description: Mass spectroscopy analysis of GBM MHC and proteome peptidome

File Name: Supplementary Data 9

Description: GSEA results from decitabine treatment of GBM cell lines
